# MicroRNA-129-5p-mediated translational repression of microglial ROCK1 leads to enhanced phagocytosis

**DOI:** 10.1016/j.jbc.2025.110293

**Published:** 2025-05-24

**Authors:** Rajib Kumar Dey, Ranjana Kumari, Roni Patra, Dharmendra Kumar Soni, Roopa Biswas, Satyakam Patnaik, Debabrata Ghosh

**Affiliations:** 1 Immunotoxicology Laboratory, Systems Toxicology Group, FEST Division, CSIR-Indian Institute of Toxicology Research (CSIR-IITR), Lucknow, Uttar Pradesh, India; 2 Academy of Scientific and Innovative Research (AcSIR), Ghaziabad, India; 3 Department of Anatomy, Physiology and Genetics, School of Medicine, Uniformed Services University of the Health Sciences, Bethesda, Maryland, USA; 4 Drug and Chemical Toxicology Group, FEST Division, CSIR-Indian Institute of Toxicology Research, Lucknow, Uttar Pradesh, India

**Keywords:** microglia, miRNA, miR-129-5p, ROCK1, arsenic, phagocytosis, p-bodies, neuroimmunology

## Abstract

ROCK1 plays an important role in phagocytosis by inducing cytoskeletal rearrangement. Although the transcriptional regulation of ROCK1 is known but its post-transcriptional regulation is underexplored. We intended to find a mechanism of microglial phagocytosis through possible post-transcriptional regulation of ROCK1. The study identified miR-129-5p as a regulator of microglial phagocytosis following exposure to an environmental stressor, arsenic, combining *in silico* analysis, mutational analysis, *in vitro* experiments, and validation in BALB/c mouse. The *in silico* analysis and *in vitro* studies with mouse primary neonatal microglia, BV2 microglia, *ex vivo* microglia, and human microglial cell line CHME3 revealed that arsenic exposure increases microglial phagocytosis. Arsenic exposure was also observed to increase the level of miR-129-5p and consequently decrease the level of ROCK1 protein. *In vitro* experiments and mutational analysis confirmed the *in silico* predicted binding site of miR-129-5p on the 3′UTR of ROCK1 and also confirmed the shuttling of ROCK1 mRNA into the cytoplasmic-processing body (p-body) in mouse microglia. Downstream to ROCK1, Rac1 has also been studied to pinpoint the partners in the signaling axis. The role of miR-129-5p in microglial phagocytosis was studied *in vitro* and validated *in vivo* in BALB/c mouse by stereotactically injecting anti-miR-129-5p and assessing the phagocytosis in *ex vivo* microglia and colocalization of Iba1 and PSD95 in brain cryosection. Finally, experiments with arsenic, anti-miR-129-5p, ROCK1 & Rac1 siRNA in various combinations confirmed the miR-129-5p→ROCK1→Rac1→Phagocytosis signaling axis. Overall, the study revealed miR-129-5p as an important regulator of microglial phagocytosis with potential implication in synaptic plasticity and neurodegenerative complications.

Microglia or brain macrophages are the resident innate immune cells in the central nervous system (CNS). They play a pivotal role in maintaining a healthy CNS milieu by providing defense against pathogens, modulating inflammation, pruning synapses, and clearing cellular debris. Phagocytosis is the key microglial function in all these cellular activities (1).

Phagocytosis is a complex process involving several key steps: recognition, engulfment, digestion, and resolution. Recognition is controlled by various receptors present on the surface of microglia. Generally, two major types of receptors, TLRs and TREM2, come into play for recognition and initiate the downstream cascade. Generally, TLRs bind to microbial pathogens, while TREM2 binds to apoptotic debris. TLRs sometimes function by forming complexes with scavenger receptors like CD14 (2). Other receptors, such as the P2Y6 receptor and complement receptors, also play roles in the clearance of degenerated or necrotic cells (3). Binding of ligands to their specific surface receptors triggers various signaling pathways, ultimately leading to cytoskeletal rearrangements and the formation of the phagosome (3), where ROCK1 plays a crucial role (4). Inhibition of ROCK1 has been shown to induce phagocytosis by macrophages (5, 6, 7) or phagocytosis of cancer cells by dendritic cells (8). Therefore, the expression of ROCK1 in microglia plays an important role in phagocytosis. Additionally, Rac1, a monomeric GTPase, plays a crucial role in membrane ruffling and phagocytosis in its active state. Rac1 activity is reported to be negatively regulated by ROCK1 (4, 9, 10), although there is no evidence of ROCK1-mediated regulation of Rac1 activity in controlling phagocytosis.

Expression of ROCK1 can be regulated transcriptionally by transcription factors or epigenetically by methylation, acetylation, or non-coding RNA. A few transcription factors have been reported to control the expression of ROCK1 in different physiological conditions. Transcription factor Sp1 positively regulates ROCK1 expression by binding to its promoter site and helps in myogenic differentiation (11). The transcription factor TFAP2C is reported to upregulate the expression of ROCK1 in colorectal cancer (11), and another transcription factor, ONECUT2, activates transcription of ROCK1 in gastric cancer (12). ROCK1 expression has also been reported to be regulated by HDACs (13).

Along with different transcription factors and HDACs, various non-coding RNAs have also been reported to regulate the expression of ROCK1. Long non-coding RNA lnc-31 helps in the translation of ROCK1 (14). Other than long non-coding RNA, various miRNAs are also involved in the regulation of ROCK1 expression in various patho-physiological conditions. Expression of ROCK1 has been observed to be regulated by miRNAs in different cancers, such as miR-124 in colorectal cancer (15), miR-124-3p in bladder cancer (16), miR-135a in gastric cancer (17), miR-144 in sarcoma (18), miR-129-5p in osteosarcoma (19) miR-148a in non-small cell lung cancer (20), miR-150 in thyroid cancer (21), miR-196a-5p in nasopharyngeal carcinoma (22), miR-584-3p in glioma (23), miR-1280 in bladder cancer (24), and miR-186 in leukemia (25). MiRNAs have also been reported to regulate the expression of ROCK1 in various physiological conditions other than cancer, such as miR-26a in lung injury (26), miR-145 in HepG2 cell proliferation (27), miR-148 in gastritis (28), and miR-148a in myogenic differentiation (29). Additionally, miR-34a and miR-124-5p have been shown to regulate phagocytosis by microglia and macrophages by targeting TREM2 and the ARP2/3 complex, respectively, but the role of ROCK1 has not been explored (30, 31).

The present study comprehensively sought to identify the mechanism of posttranscriptional regulation of ROCK1 and its impact on microglial phagocytosis. We examined phagocytosis in BV2 microglia and *ex vivo* microglia (microglia isolated from control and arsenic-exposed animals) as well as human microglial cell line CHME3. Expression of ROCK1 has also been checked in BV2 microglia, *ex vivo* microglia, neonatal primary microglia, CHME3 cells following *in vitro* arsenic exposure, and in the hippocampus of arsenic exposed mouse. Using the TaqMan Low-Density Array (TLDA) for miRNAs and various *in silico* tools, we predicted specific miRNAs that could potentially modulate ROCK1 expression. The binding of miRNA to ROCK1 was confirmed by a luciferase assay using mutant constructs of the 3′-UTR of ROCK1. ROCK1 mRNA degradation was assessed in the presence or absence of arsenic and pre-miR. The role of miRNA in microglial phagocytosis was confirmed using anti-miR (miRNA inhibitor). The study was extended to an *in vivo* setting, where the miRNA was knocked down by injecting its specific anti-miR into the brain using stereotactic surgery, followed by assessing phagocytosis by *ex vivo* microglia, and colocalization of the microglia-specific marker Iba1 and neuronal Post Synaptic Density protein 95 (PSD95). Overall, the findings revealed that miR-129-5p controls microglial phagocytosis by posttranscriptional regulation of ROCK1.

## Results

### Microglial phagocytosis is increased by arsenic

**Figure 1:**
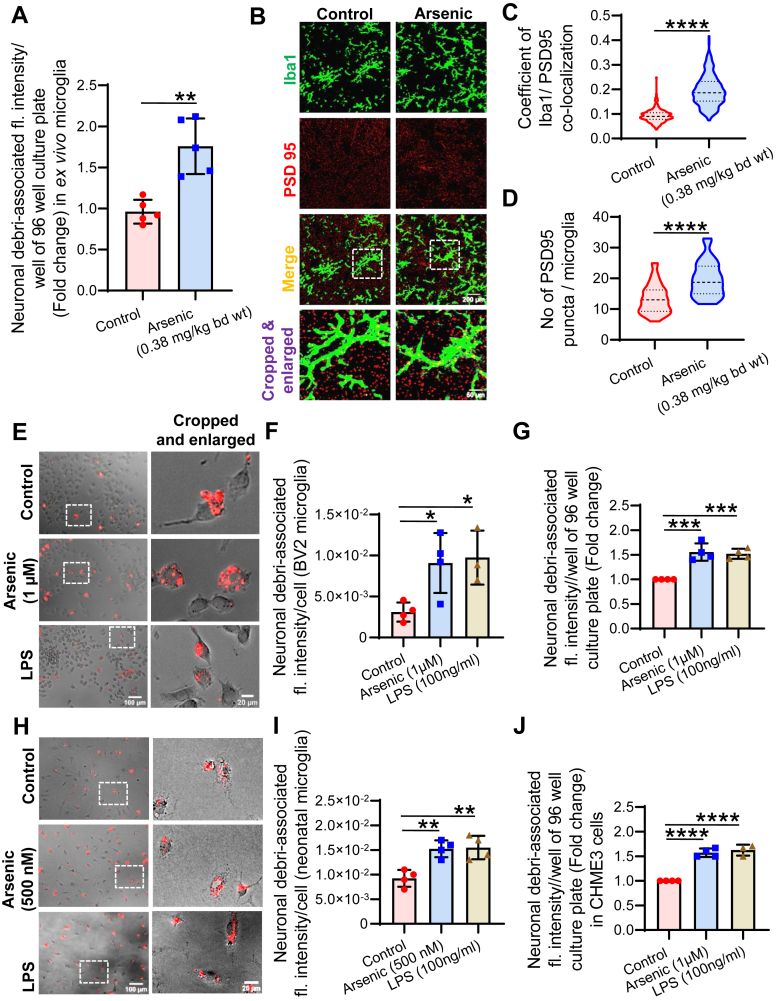
**Effect of arsenic stress on microglial phagocytosis.** Pregnant dams were exposed to sodium arsenite (arsenic) from gestational day (GD) 05 till post natal day (PND) 22 (till weaning of pups). *A*, primary microglia (*ex vivo* microglia) were isolated from brains and phagocytic activity was checked using CM-DiI dye loaded neuronal debri. n = 10 animals (microglia from 2 animals pooled together). *B*, brains were isolated from the animals and processed for cryosectioning and co-immunostaining using Iba1 and PSD95. n = 5 animals (3 sections in hippocampus region of each animal, 10–12 fields (100× magnification) in each section) scale bar = 200 μm for uncropped images and 50 μm for cropped images. *C*, coefficient of Iba1/PSD95 colocalization and (*D*) No of PSD95 puncta/microglia was measured in ImageJ and represented as violin plot. n = 5 animals (3 sections in hippocampus region of each animal, 6–8 fields (100× magnification) in each section). BV2 microglia were seeded in 96-well culture plated and tested for phagocytosis of neuronal debri. *E*, representative images of BV2 with neuronal debri (*red*). *F*, neuronal debri-associated fluorescence/cell measured in ImageJ and represented as hybrid scatter-bar graph. *G*, BV2 microglia were seeded in 96 well culture plated, incubated with neuronal debri, washed and the total neuronal debri-associated fluorescence/well was measured in a micro plate reader and represented as hybrid scatter-bar graph. Similar to *G* phagocytosis of neuronal debri was assessed for (*H* & *I*) mouse primary neonatal microglia and (*J*) human microglia, CHME3. n = 3 to 4 independent *in vitro* experiments. Bar graphs represent mean ± SD. For comparing control and arsenic-exposed group Mann-whitney test was performed and “One-way ANOVA” was used for comparing more than two groups followed by *post hoc* analysis by “Tukey’s test”. “*p*” denotes the level of significance in comparison to control; ∗*p* < 0.05, ∗∗*p* < 0.01, ∗∗∗*p* < 0.001, ∗∗∗∗*p* < 0.0001; ns, non-significant.

Microglial phagocytosis is associated with its activation status. Earlier, we showed that arsenic exposure activates microglia; therefore, the effect of arsenic on microglial phagocytosis was measured with the intention of finding out the underlying mechanism. Pregnant BALB/c females were exposed to arsenic (0.38 mg/kg bd wt) from gestational day 5 (GD5) till the weaning of the pups (PND22). Following weaning, pups were sacrificed, microglia were isolated (*ex vivo* microglia), and a phagocytosis assay was performed using Dil-dye stained neuronal debri. Arsenic-exposed *ex vivo* microglia showed higher phagocytosis compared to the control group (∼1.7 fold) (Fig. 1*A*). As supportive evidence, colocalization of microglial marker, Iba1, and neuronal marker, PSD95, was performed in cryo-section of the hippocampus (Fig. 1*B*). Coefficient of Iba1/PSD95 colocalization (Fig. 1*C*) and number of PSD95 puncta/microglia (Fig. 1*D*) were calculated from the Iba1/PSD95 colocalized images. It was observed that a higher coefficient of colocalization and more PSD95 puncta/microglia were observed in the arsenic-exposed group (Fig. 1, *B*–*D*). We have extended our study to *in vitro* settings by using mouse microglial cell line BV2, primary neonatal mouse microglia, and human microglial cell line CHME3 to check the commonality of phagocytic response in exposure to arsenic among the microglia of different origins. Microglia were exposed to arsenic in a cell culture plate, and 3h before the completion of the experiment (72 h), CM-Dil-dye-stained neuronal debri were added to the culture and assessed for phagocytosis. It was observed that BV2 microglia (Fig. 1, *E*–*G*), primary neonatal microglia (Fig. 1, *H* and *I*), and CHME3 (Fig. 1*J*) cells showed significantly higher phagocytic potential following arsenic exposure compared to unexposed cells. Therefore, it has been evident that arsenic exposure increases phagocytosis unanimously across microglia of different origins.

### Microglial phagocytosis is associated with ROCK1 expression

**Figure 2:**
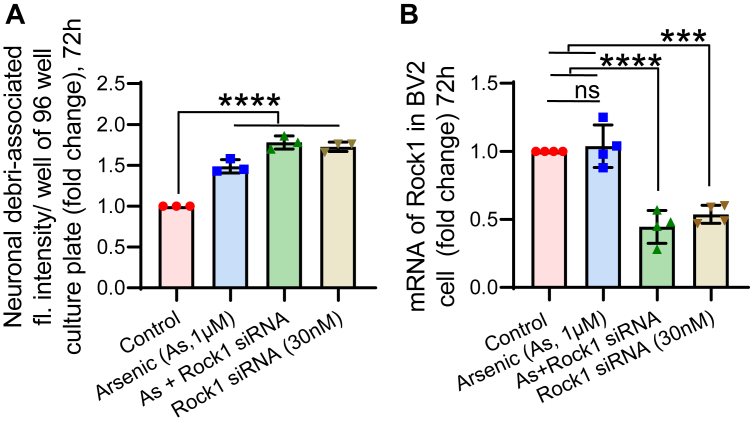
**Effect of inhibition of ROCK1 on microglial phagocytosis.** BV2 microglia were seeded in 6 well culture plate and treated with arsenic and ROCK1 siRNA, followed by (*A*) phagocytosis assay. n = 3 independent *in vitro* experiment, and (*B*) PCR to detect ROCK1 mRNA level. n = 4 independent *in vitro* experiment. Bar graphs represent mean ± SD. For comparing more than two groups “One-way ANOVA” was used followed by *post hoc* analysis by “Tukey’s test”. “*p*” denotes the level of significance in comparison to control; ∗*p* < 0.05, ∗∗*p* < 0.01, ∗∗∗*p* < 0.001, ∗∗∗∗*p* < 0.0001; ns, non-significant.

Phagocytosis is a tightly controlled process that involves the movement of the cell, thereby intimately associated with the cytoskeletal rearrangement. Rho-associated coiled-coil-containing protein kinase 1 or ROCK1 is a regulator of F-actin cytoskeleton which promotes contractile force and helps in phagocytosis. Therefore, we wanted to check the involvement of ROCK1 in case of arsenic-induced phagocytosis. BV2 cells were treated with various combinations of arsenic and ROCK1 siRNA (30 nM) for 72 h followed by phagocytosis assay and qRT-PCR. It was observed that phagocytosis was increased in arsenic-exposed group (∼1.5 fold) compared to the control group. At the same time, a significant increase was also observed in only ROCK1 siRNA-treated group (∼1.7 fold). Similar to individual treatment cotreatment of arsenic and ROCK1 siRNA also showed increased phagocytosis (∼1.8 fold) compared to control group (Fig. 2*A*). Along with phagocytosis assay, expression of ROCK1 was also checked. It was observed that the level of ROCK1 mRNA was decreased in siRNA (∼0.45 fold) alone and siRNA & arsenic co-exposed group (∼0.44 fold) compared to the control group. Interestingly, the level of mRNA was not decreased in only arsenic-exposed group (∼1.04 fold) (Fig. 2*B*). Therefore, it has been evident that inhibition of ROCK1 plays an important role in microglial phagocytosis and there is an indication that arsenic-mediated inhibition of ROCK1 is not regulated transcriptionally.

### ROCK1 mRNA and protein is differentially regulated by arsenic exposure in microglia

**Figure 3:**
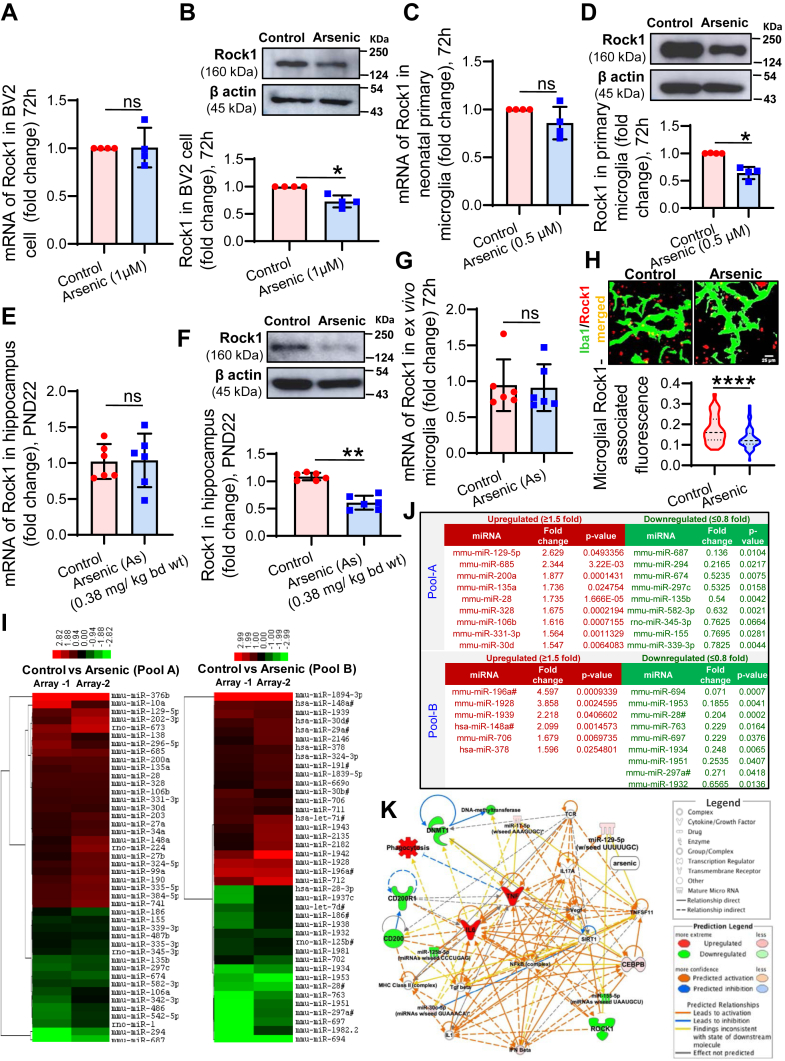
**Effect of arsenic on the level of mRNA and protein of ROCK1.** BV2 microglia were seeded in 6 well culture plates and treated with arsenic for 72 h. Following treatment total mRNA and protein were isolated for RT-PCR and Western blot analysis. The level of ROCK1 mRNA and protein were measured in (*A* and *B*) BV2 microglia (n = 4 independent experiment), (*C* and *D*) neonatal primary microglia (n = 4 independent experiment). *E* and *F*, hippocampi were isolated from the gestationally arsenic-exposed (from GD05 to PND22) pups, the level of ROCK1 mRNA and protein were measured (n = 6 animals/group). *G* and *H*, primary microglia were isolated from gestationally exposed pups (PND22) and the level of ROCK1 mRNA measured using qRT-PCR, in hippocampus cryosection the level of ROCK1 was measured by colocalizing ROCK1 and Iba1. n = 12 animals/group (microglia from 2 animals pooled together) for PCR and n = 4 animals/group for Iba1 (*green*) & ROCK1 (*red*) colocalization. Scale bar: 25 μm (*I*) Primary neonatal microglia were treated with arsenic *in vitro* for 72 h, and the TaqMan Low Density Array (TLDA) was run to detect the changes in global miRNA profile (n = 2), the fold change in the level of miRNA was presented as a heatmap. *J*, MiRNAs upregulated more than 1.5-fold and down regulated more than 0.8-fold was presented in the table (the *I* and *J* have been reused from our earlier publication, JBC Volume 298, Issue 1, 2022, 101,521; https://doi.org/10.1016/j.jbc.2021.101521 with permission). *K*, network generated by ingenuity pathway analysis (IPA) tool with up- and down-regulated genes and miRNA in arsenic-treated microglia indicated a possible relationship between ROCK1 and miR-129-5p. The symbols used in the network map are shown in the legend. ‘n’ denotes the number of animals for *in vivo* studies and number of independent experiments for *in vitro* studies. Bar graphs represent mean ± SD. For comparing control and arsenic-exposed group Mann-whitney test was performed. “*p*” denotes the level of significance in comparison to control; ∗*p* < 0.05, ∗∗*p* < 0.01, ∗∗∗*p* < 0.001, ∗∗∗∗*p* < 0.0001; ns, non-significant.

We already have an indication from the earlier result section that expression of ROCK1 is not regulated transcriptionally following arsenic exposure. With an intention to confirm the regulation of its expression, qRT-PCR and Western blot analysis were employed. Expression of ROCK1 was checked in the BV2 cell line, neonatal primary microglia, hippocampus tissue, and *ex vivo* microglia to show uniformity of response across the different cell systems. For *in vitro* exposure, BV2 (Fig. 3, *A* and *B*) and primary neonatal microglia (Fig. 3, *C* and *D*) were exposed to arsenic for 72 h, and levels of ROCK1 mRNA and protein were measured. It was observed that the level of mRNA was not altered significantly following arsenic exposure in BV2 (∼1.01 fold) (Fig. 3*A*) as well as in neonatal primary microglia (∼0.86 fold) (Fig. 3*C*), whereas, protein level was significantly decreased both in BV2 (∼0.73 fold) (Fig. 3*B*) and neonatal primary microglia (∼0.71 fold) (Fig. 3*D*). Similarly, the level of ROCK1 mRNA (∼1.03 fold) was not altered in hippocampus tissue (Fig. 3*E*), but the level of protein (∼0.61 fold) was significantly decreased in arsenic-exposed group (Fig. 3*F*). ROCK1 mRNA was also checked in microglia isolated from control and arsenic-exposed (0.38 mg/kg bd wt) animals (*ex vivo* microglia); it was observed to follow a similar pattern to other exposure groups (Fig. 3*G*). As the yield of *ex vivo* microglia is very low, we have checked the level of ROCK1 in microglia by colocalizing ROCK1 and Iba1 in cryo-sections of the hippocampus of control and arsenic-exposed animals (0.38 mg/kg bd wt). Significantly decreased ROCK1-associated immunofluorescence was observed in the arsenic-exposed group (Fig. 3*H*). Therefore, it is evident that ROCK1 expression is regulated post-transcriptionally by arsenic. MicroRNAs are the major players in post-transcriptional regulation of gene expression. Therefore, TaqMan Low Density Array (TLDA) was run to find if any miRNA is involved in the process in primary microglia following *in vitro* arsenic (0.5 μM) treatment for 72 h. MiRNAs with significant differential expression are presented in a heatmap (Fig. 3*I*). A total of 15 significantly upregulated (≥1.5 fold) and 18 significantly downregulated (≤0.8 fold) miRNAs were identified (Fig. 3*J*) (the Fig. 3, *I* and *J* have been reused from our earlier publication, JBC Volume 298, Issue 1, 2022, 101,521; https://doi.org/10.1016/j.jbc.2021.101521 with permission). We uploaded the list of miRNA and mRNA of our interest into Ingenuity pathway analysis and built a network to identify any possible relationships between various molecules. We could not find any direct link between ROCK1 and miR-129-5p (Fig. 3*K*).

### MicroRNA-129-5p is involved in the post-transcriptional regulation of ROCK1 expression

**Figure 4:**
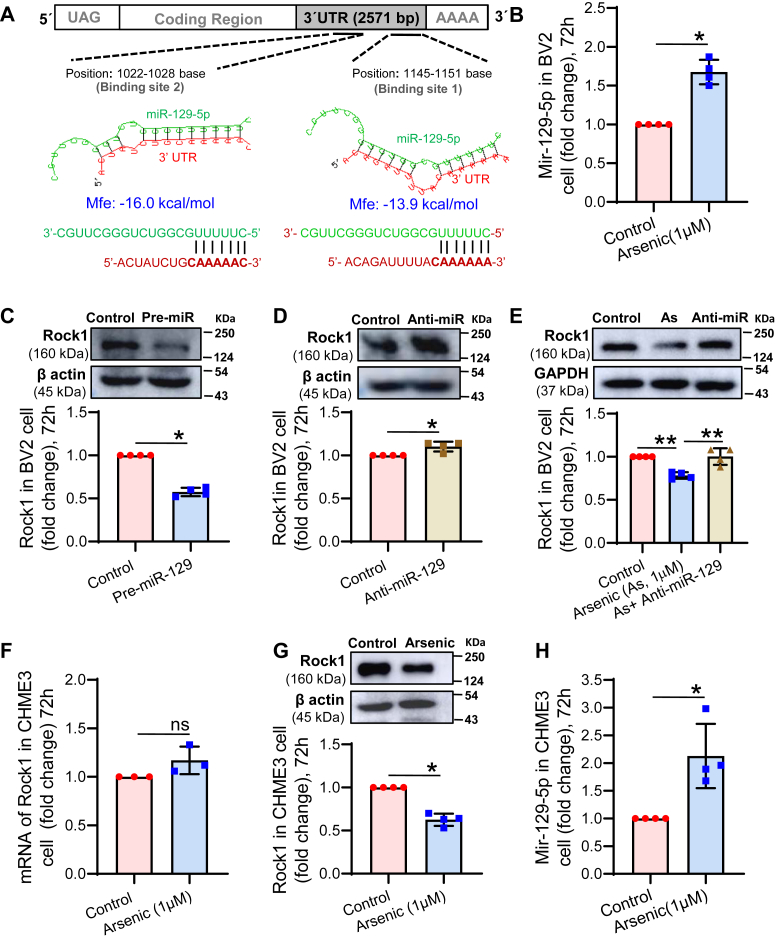
**Effect of arsenic and miR-129-5p on the expression of ROCK1.***A*, *in silico* analysis using TargetScan and RNAhybrid predicted the two putative miR-129-5p binding sites (1022–1028 bases and 1145–1151 bases) in the 3′ UTR of mouse ROCK1 mRNA. *B*, *in vitro* arsenic exposure increased the level of miR-129-5p in BV2 microglia. Level of ROCK1 following exposure to (*C*) pre-miR-129-5p and (*D*) anti-miR-129-5p. *E*, arsenic-induced decreased level of ROCK1 was reversed following anti-miR-129-5p treatment. Human microglia cell line CHME3 cells were treated with arsenic for 72 h and the level of (*F*) ROCK1 mRNA, (*G*) ROCK1 protein, and (*H*) miR-129-5p were checked. n = 3 to 4 independent experiments. All the quantitative data were represented hybrid scatter-bar graph. ‘n’ denotes the number of independent experiment for *in vitro* studies. Bar graphs represent mean ± SD. For comparing control and arsenic-exposed group Mann-whitney test was performed. “*p*” denotes the level of significance in comparison to control; ∗*p* < 0.05, ∗∗*p* < 0.01, ∗∗∗*p* < 0.001, ∗∗∗∗*p* < 0.0001; ns, non-significant.

*In silico* analysis with TargetScan and RNA hybrid showed that miR-129-5p targets ROCK1 by binding to its 3′-UTR. Two putative binding sites of miR-129-5p (bp 1022–1028 and 1145–1151) were identified in the 3′-UTR of mouse ROCK1 mRNA (Fig. 4*A*). Following TLDA analysis, individual analysis showed that the level of miR-129-5p was significantly elevated in the BV2 microglia exposed to arsenic *in vitro* (∼1.67 fold) (Fig. 4*B*). Thus, it was confirmed that exposure to arsenic increases the level of miR-129-5p. Next, the role of miR-129-5p was studied in the regulation of ROCK1 expression. The dose of pre- and anti-miR-129-5p was adopted from our earlier publication (32). For pre-miR, 10 nM and anti-miR, 100 nM were used for transfection studies. The ROCK1 protein levels decreased significantly (∼0.57 fold) in BV2 microglia exposed to pre-miR-129 for 72 h *in vitro* (Fig. 4*C*), and the exposure to anti-miR-129-5p increased the expression (∼1.1 fold) (Fig. 4*D*). A similar effect of anti-miR-129-5p was observed in arsenic-exposed microglia where anti-miR-129-5p reversed the arsenic-induced downregulation of ROCK1 (∼0.78 fold in arsenic and ∼1.0 fold in arsenic + anti-miR group) (Fig. 4*E*). Reversal of arsenic-induced downregulation of ROCK1 by anti-miR supports our notion of post-transcriptional regulation of ROCK1 by miR-129-5p. To check the commonality of miR-129-5p function across the species, human microglial cell line, CHME3 cells, were exposed to arsenic for 72 h and checked for the mRNA and protein levels. It was observed that the level of ROCK1 mRNA was not altered significantly (∼1.17 fold) in arsenic arsenic-treated group compared to the control group (Fig. 4*F*), whereas the protein level was decreased significantly following arsenic exposure (∼0.63 fold) (Fig. 4*G*). Increased expression of the miR-129-5p (∼2.13 fold) following *in vitro* arsenic exposure in CHME3 cells (Fig. 4*H*) also supports the involvement of arsenic-induced upregulation of miR-129-5p across the species. Therefore, the involvement of miR-129-5p in the post-transcriptional regulation of ROCK1 expression has been evident from the data in Figure 4.

### MicroRNA-129-5p binds to 3′ UTR of ROCK1 and guides it to P-bodies

**Figure 5:**
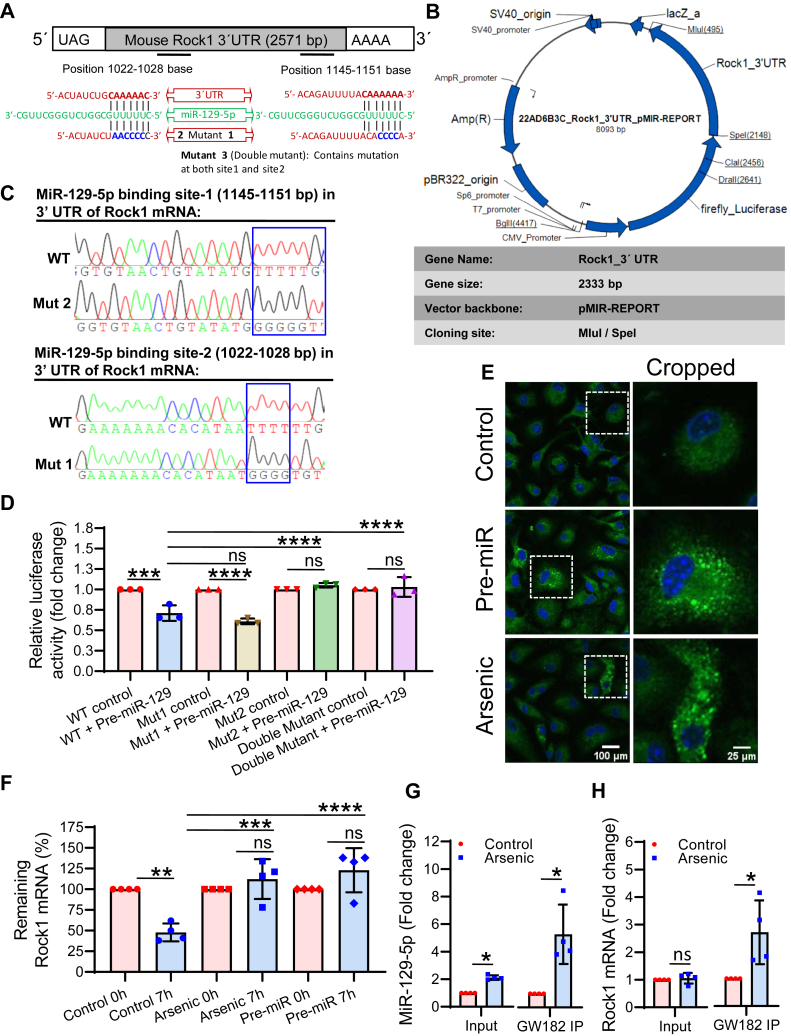
**Binding site of miR-129-5p in 3′-UTR of ROCK1.***A*, Sequence alignment of ROCK1 3′-UTR, miR-129-5p seed sequence and mutant sequence (Mutants 1 & 2) of 3′-UTR. *B*, detail map of ROCK1 3′UTR luciferase reporter plasmid. *C*, sequence chromatograms showing the WT and mutant sequence of site 1 and site 2. *D*, luciferase reporter assay using mouse ROCK1 3′-UTR constructs (pMIR-WT, pMIR-Mut1, pMIR-Mut2 and pMIR-Mut3[ double mutant]) cloned in pMIR-REPORT vector confirmed that both the predicted binding sites are involved in miR-129-5p-mediated repression of ROCK1 (n = 3 independent experiment). *E*, P-bodies were stained with GW182 antibody in BV2 microglia following pre-miR-129 and arsenic exposure for 72 h. It showed increased puncta formation in the cytoplasm. Scale bar: 100 μm for uncropped images and 25 μm for the cropped images. *F*, microglia were treated with arsenic for 72 h followed by actinomycin D (1.5 μg/ml) treatment for 0, 7 h. Cells were harvested, RNA isolated, and run for real-time PCR to detect ROCK1 mRNA. Arsenic protected ROCK1 mRNA from degradation (n = 4 independent experiment). To detect the presence of miR-129-5p and ROCK1 mRNA in p-bodies, GW182 was over expressed in BV2 cells, simultaneously treated with arsenic and the level of (*G*) miR-129-5p (data for *G* has been reused from our earlier publication, JBC Volume 298, Issue 1, 2022, 101,521; https://doi.org/10.1016/j.jbc.2021.101521 with permission), and (*H*) ROCK1 mRNA were measured in GW182 IP samples. Levels of both miR-129-5p and ROCK1 mRNA in the GW182 IP samples were found to be significantly high (n = 4). “n” denotes the number of independent study for *in vitro* experiments. Bar graphs represent mean ± SD. For comparing control and arsenic-exposed group Mann-whitney test was performed and “One-way ANOVA” was used for comparing more than two groups followed by *post hoc* analysis by “Tukey’s test”. “*p*” denotes the level of significance in comparison to control; ∗*p* < 0.05, ∗∗*p* < 0.01, ∗∗∗*p* < 0.001, ∗∗∗∗*p* < 0.0001; ns, nonsignificant.

*In silico* analysis predicted two binding sites of miR-129-5p in the 3′ UTR of mouse ROCK1 mRNA. To confirm the binding of miR-129-5p to the two putative binding sites (binding site 1: 1145–1151 and binding site 2: 1022–1028) in the 3′ UTR of mouse ROCK1 mRNA, WT and mutant luciferase constructs were generated using pMIR-REPORT vector (Fig. 5, *A* and *B*). For site 1 sequence of 3′ UTR were changed from CAAAAA to AACCCC and for site 2 sequences were changed from CAAAAA to CACCCC by mutagenesis of the WT sequences and confirmed by sequencing analysis (Fig. 5*C*). Binding of miRNA to the WT and mutated sequence of 3′ UTR of mouse ROCK1 mRNA was validated by luciferase assay (Fig. 5*D*). HEK293 cells pre-treated with or without pre-miR-129-5p for 8 h were transfected with wild-type (WT) and mutant (Mut) pMiR-REPORT constructs of 3′ UTR of ROCK1 mRNA. 48 h post-transfection, luciferase assay was performed, and it was observed that the luciferase activity had significantly decreased in the WT pre-treated with miRNA (∼0.71 fold). A similar response was observed in Mut1 (∼0.61 fold). Interestingly, Mut2 and in double mutant (Mut3), the luciferase activity was observed to be reversed (Mut2: ∼1.1 fold and Mut3: 1.0-fold) compared to the WT pre-miR-129 group (Fig. 5*D*). It has been evident from the luciferase activity that predicted binding site 1 (mut1) does not play any role in the binding of miR-129-5p. Whereas, the luciferase activity in mut2 and double mutant (mut3) confirms site 2 to be essential for miR-129-5p binding in the 3′ UTR of ROCK1 mRNA. Therefore, only the binding site 2 is involved in the post transcriptional regulation ROCK1 expression. When microRNA binds to mRNA, either it induces its degradation or guides it to cytoplasmic processing bodies (P-bodies), where it became inaccessible to the translational machinery and induces translational repression. We studied whether there was an increased formation of P-bodies by immunostaining of GW182, which is an essential component for P-bodies. Both arsenic and pre-miR treatment induced the formation of P-bodies in microglia, visible as puctate green fluorescent body in the cytoplasm (Fig. 5*E*). As there is formation of P-bodies, there is possibility that ROCK1 mRNA is guided to P-bodies and as a result become inaccessible to the degradation machinery. Therefore, to test this possibility, the decay of ROCK1 mRNA was studied following P-body induction either by pre-miR or arsenic. The time required for 50% degradation of ROCK1 mRNA in microglia was determined to be 7 h (data not shown) which was used in subsequent studies. Following P-body induction, cells were treated with actinomycin D (Act D), harvested at 0 and 7 h and the level of remaining mRNA was measured by qRT-PCR (Fig. 5*F*). Approximately 50% (∼52%) mRNA decay was observed in control group after 7 h of Act D treatment. Interestingly, no significant alteration was observed in arsenic (∼112%) and pre-miR (∼123%) treated group following Act D treatment (Fig. 5*F*). Protection of ROCK1 mRNA decay in arsenic as well as in pre-miR-treated groups possibly due to its inclusion into the P-bodies. Therefore, exact localization of ROCK1 mRNA was confirmed by pull-down of P-bodies and detecting the level of miR-129-5p as well as ROCK1 mRNA in it.

GW182 was over expressed in arsenic-exposed microglia. P-bodies were immune-precipitated (IP) with the GW182 antibody, and the total RNA was isolated. The level of miR-129-5p and ROCK1 mRNA were evaluated by qRT-PCR. Significant enrichment of miR-129-5p (∼5.32 fold) (Fig. 5*G*) (data for Fig. 5*G* has been reused from our earlier publication, JBC Volume 298, Issue 1, 2022, 101,521; https://doi.org/10.1016/j.jbc.2021.101521 with permission) and ROCK1 mRNA (∼2.68 fold) (Fig. 5*H*) was also observed in the IP samples of the arsenic-treated group. The enrichment of miR-129-5p and ROCK1 mRNA in the IP samples in arsenic-treated groups confirms that miR-129-5p binds to ROCK1 mRNA and brings it to the p-body, thereby inducing translation repression.

### MicroRNA-129-5p is involved in microglial phagocytosis

**Figure 6:**
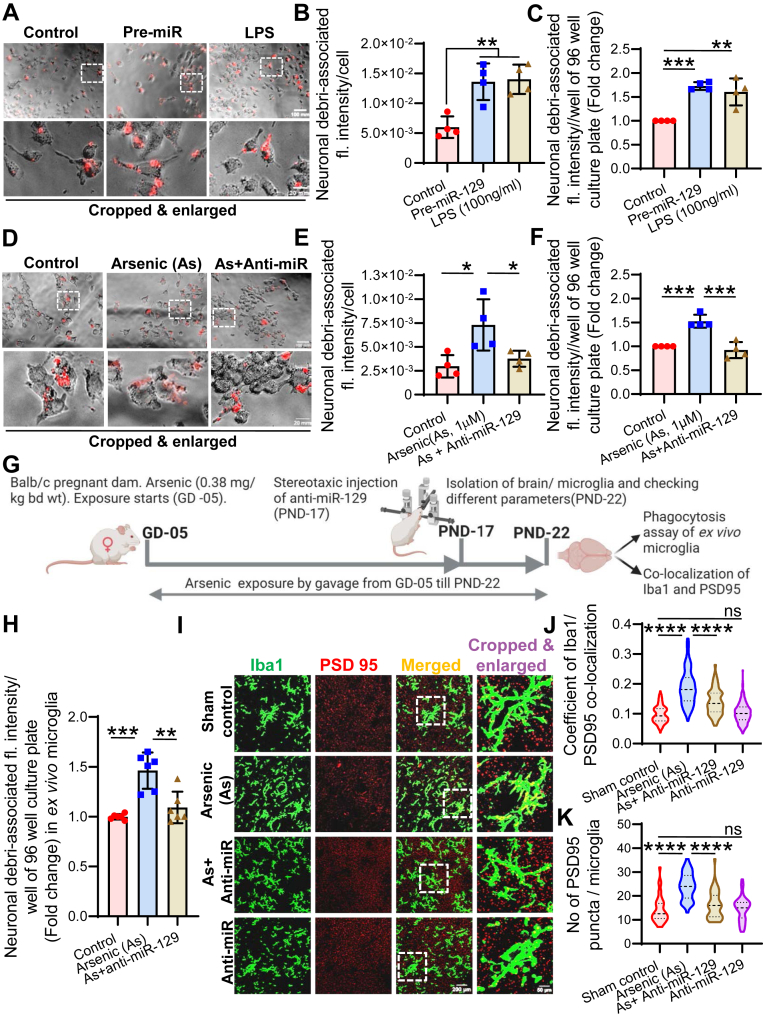
**Effect of *in vitro* and *in vivo* inhibition of miR-129-5p on microglial phagocytosis.** BV2 microglia were seeded in 96 well culture plates, treated with either pre-miR-129-5p or LPS for 72 h and checked for phagocytosis. *A*, representative images of BV2 with neuronal debri (*red*). *B*, neuronal debri-associated fluorescence/cell measured in ImageJ and represented as hybrid scatter-bar graph. *C*, BV2 microglia were seeded in 96 well culture plated, incubated with neuronal debri, washed and the total neuronal debri-associated fluorescence/well was measured in a micro-plate reader and represented as hybrid scatter-bar graph. In another set of experiment, BV2 microglia were seeded in 96 well culture plate, treated with either arsenic or anti-miR-129-5p for 72 h and checked for phagocytosis. *D*, representative images of BV2 with neuronal debri (*red*). *E*, neuronal debri-associated fluorescence/cell measured in ImageJ and represented as hybrid scatter-bar graph. scale bar: 100 μm for uncropped images and 20 μm for cropped images in *A* and *D*. *F*, BV2 microglia were seeded in 96 well culture plated, incubated with neuronal debri, washed and the total neuronal debri-associated fluorescence/well was measured in a micro-plate reader and represented as hybrid scatter-bar graph. n = 3 to 4 independent *in vitro* experiments. *G*, to check the impact of *in vivo* administration of anti-miR-129-5p, pregnant dams were exposed to arsenic from gestational day (GD) 05 till post natal day (PND) 22 (till weaning of pups). *H*, primary microglia (*ex vivo* microglia) were isolated from brains and phagocytic activity was checked using dill-dye loaded neuronal debri. n = 12 animals (microglia from 2 animals pooled together). *I*, brains were isolated from the animals and processed for cryosectioning and co-immunostaining using Iba1 and PSD95. Scale bar: 200 μm for uncropped images and 50 μm for cropped images. *J*, coefficient of Iba1/PSD95 colocalization. n = 4 to 5 animals (3 sections in hippocampus region of each animal, 10–12 fields (100× magnification) in each section) and (K) Number of PSD95 puncta/microglia was measured in ImageJ and represented as violin plot. n = 4 to 5 animals (3 sections in hippocampus region of each animal, 6–8 fields (100× magnification) in each section). Bar graphs represent mean ± SD. For comparing more than two groups “One-way ANOVA” was used followed by *post hoc* analysis by “Tukey’s test”. “*p*” denotes the level of significance in comparison to control; ∗*p* < 0.05, ∗∗*p* < 0.01, ∗∗∗*p* < 0.001, ∗∗∗∗*p* < 0.0001; ns, non-significant.

We have already shown that arsenic exposure increases microglial phagocytosis where ROCK1 plays an important role. It has also been proven that arsenic controls the expression of ROCK1 epigenetically through miR-129-5p. Therefore, the role of miR-129-5p in microglial phagocytosis was studied through *in vitro* and *in vivo* experiments. Microglia were exposed to either pre-miR-129 or LPS, incubated for 72 h and 18 h respectively followed by phagocytosis assay. An increased phagocytosis was observed in pre-miR-129-exposed group, similar to LPS-exposed group, which is a known to induce phagocytosis (Fig. 6, *A*–*C*). In previous result section, it has been shown that arsenic exposure increases the phagocytosis (Fig. 1) and also increases the level of miR-129-5p (Fig. 4*B*). Therefore, the effect of anti-miR-129-5p treatment on the phagoytic activity of arsenic-exposed cells was studied. It was observed that anti-miR-129-5p treatment could bring down the arsenic-induced increased phagocytosis in microglia (Fig. 6, *D*–*F*). The role of miR-129-5p in microglial phagocytosis was further validated in microglia isolated from control, arsenic and arsenic & anti-miR-129-5p co-treated group (*ex vivo* microglia) as shown in scheme (Fig. 6*G*). An increased phagocytosis was observed in arsenic exposed group, which decreased significantly following *in vivo* administration of anti-miR-129-5p into the brain of experimental animals through stereotactic injection (Fig. 6*H*). In support of the modulation of phagocytosis by miR-129-5p in *ex vivo* microglia, microglial marker, Iba1 and neuronal marker, PSD95 was co-localized in cryosection of hippocampus of experimental animals (Fig. 6*I*). From the images used for colocalization in Figure 6*I*, coefficient of Iba1/PSD95 colocalization (Fig. 6*J*) and number of PSD95 puncta/microglia (Fig. 6*K*) were calculated and expressed as violin plot. A higher coefficient of colocalization and more PSD95 puncta/microglia was observed in arsenic-exposed group, which were reduced by administration of anti-miR-129-5p in the brain. Anti-miR-129-5p alone-treated group did not show any significant alteration compared to control group (Fig. 6, *J* and *K*). Altogether, the data in Figure 6 confirm the involvement of miR-129-5p in microglial Phagocytosis.

### Phagocytic signal follows the miR-129-5p→ROCK1→Rac1→phagocytosis axis

**Figure 7:**
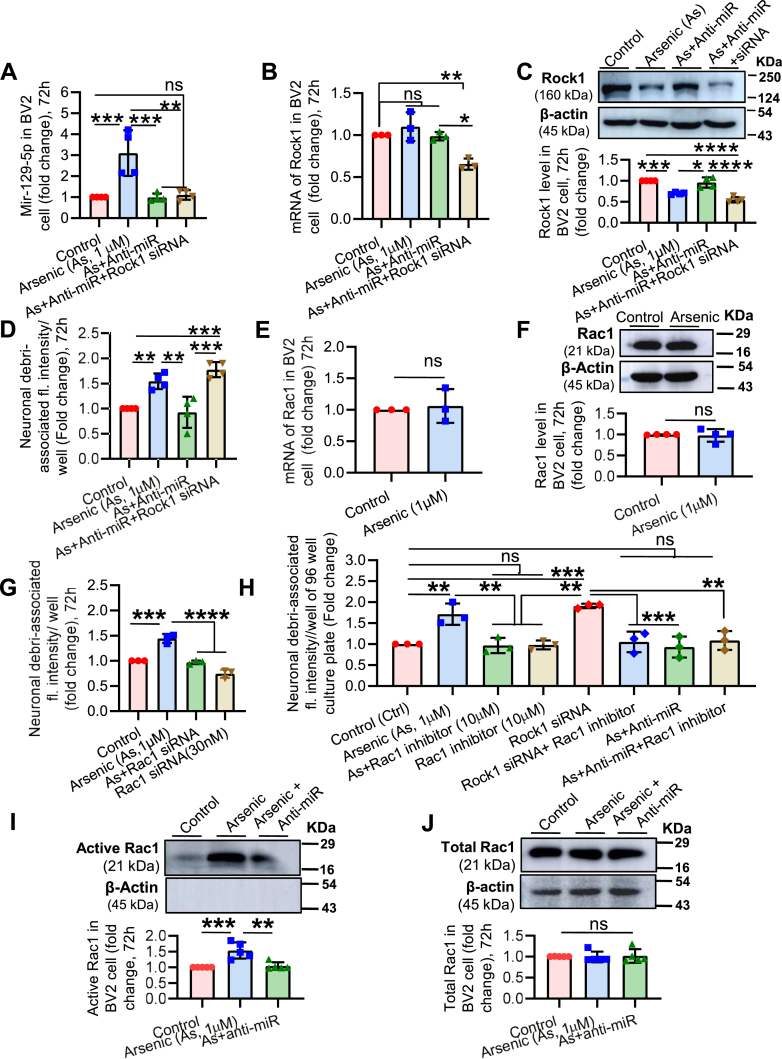
**Effect of inhibition of ROCK1, miR-129-5p and Rac1 on microglial phagocytosis.** BV2 microglia were treated *in vitro* with various combination of arsenic, anti-miR and ROCK1 siRNA and cultured for 72 h. *A*, cells were harvested and the levels of miR-129-5p were checked in control and different treatment groups. *B*, the levels of ROCK1 mRNA were also checked using qRT-PCR. *C*, Levels of ROCK1 were checked using Western blot analysis in various experimental groups. *D*, phagocytic potential was also checked in control and treated BV2 cells. At 69th h of incubation CM-Dil dye-tagged neuronal debri were added to different treatment groups of BV2 cells and incubated further for 3 h and assessed for phagocytosis. *E*, level of Rac1 mRNA and (*F*) Rac1 protein were found to be unaffected by arsenic exposure. *G*, involvement of Rac1 in phagocytosis was determined by using siRNA. Rac1 siRNA was observed to reverse the arsenic-induced enhanced phagocytosis. *H*, phagocytosis assay was performed in exposure to various combinations of arsenic, anti-miR-129-5p, Rac1 inhibitor and ROCK1 siRNA to to confirm the signaling axis. Effect of arsenic and anti-miR-129-5p on the level of active Rac1 was also detected in BV2 cells, (*I*) the image of the Western blot represents the level of active Rac1 in the pull-down sample (equal volume of the pull down sample loaded in each lane). *J*, the image of Western blot represents the level of total Rac1 in the whole cell lysate. The band intensity of the blots were quantitated and represented as scatter bar graph. n = 3 to 5 independent *in vitro* experiments. All the quantitative data were represented as hybrid scatter-bar graph. Bar graphs represent mean ± SD. For comparing control and arsenic-exposed group Mann-whitney test was performed and “One-way ANOVA” was used for comparing more than two groups followed by *post hoc* analysis by “Tukey’s test”. “*p*” denotes the level of significance in comparison to control; ∗*p* < 0.05, ∗∗*p* < 0.01, ∗∗∗*p* < 0.001, ∗∗∗∗*p* < 0.0001; ns, non-significant.

We have previously demonstrated that ROCK1 is involved in arsenic-induced microglial phagocytosis. We have also shown that miR-129-5p regulates microglial phagocytosis through the post-transcriptional regulation of Rock1. To investigate whether the phagocytic signal operates through the miR-129-5p→ROCK1→Rac1→phagocytosis axis, BV2 cells were exposed to various combinations of arsenic, anti-miR, ROCK1 siRNA, and different parameters were measured. The level of miR-129-5p increased in the arsenic-exposed group, which was brought back to control levels by anti-miR treatment. Co-treatment with arsenic, anti-miR, and ROCK1 siRNA did not show any significant changes compared to the control (Fig. 7*A*). The level of ROCK1 mRNA was also examined; except for the arsenic, anti-miR, and siRNA co-treatment group, no other group showed changes in ROCK1 mRNA levels compared to the control group (Fig. 7*B*). Interestingly, the level of ROCK1 protein did not follow the same pattern as ROCK1 mRNA. The arsenic-exposed group showed decreased ROCK1 protein levels, which were reversed by anti-miR treatment. In the arsenic, anti-miR, and siRNA co-treatment group, the effect of siRNA was evident, as shown by the decreased ROCK1 protein levels (Fig. 7*C*). The impact of altered expression of miR-129-5p and ROCK1 on microglial phagocytosis was also tested. As observed earlier, the level of ROCK1 and the microglial phagocytosis showed an inverse relationship. Phagocytosis was higher in the arsenic-exposed group, where ROCK1 levels were low, and decreased phagocytosis was observed in the arsenic and anti-miR co-exposed group, where ROCK1 levels were higher (Fig. 7*D*). These data corroborate that miR-129-5p post-transcriptionally regulates ROCK1, which in turn controls microglial phagocytic function.

Next, we focused on investigating the involvement of Rac1 in miR-129-5p-mediated regulation of microglial phagocytosis through ROCK1. Rac1 is a potential downstream target of ROCK1, which contributes to membrane ruffling and phagocytosis (9, 10). Interestingly, a negative feedback loop exists between RhoA, ROCK1, and Rac1 (33), despite there is no evidence of ROCK1-mediated regulation of Rac1 activity in controlling phagocytosis. To explore this research gap, we first check Rac1 expression in BV2 cell following arsenic exposure. However, we did not find any significant change both in Rac1 mRNA and protein level, indicating no impact of arsenic on Rac1 gene expression (Fig. 7, *E* and *F*). Interestingly, co-treatment of BV2 cell with arsenic and Rac1 siRNA (30 nM) showed a significant decrease in phagocytosis compared to only arsenic-exposed group, suggesting potential involvement of Rac1 downstream to ROCK1 in regulating phagocytosis in arsenic-exposed microglia (Fig. 7*G*). Similar observation was noted when Rac1 activity was inhibited using Rac1 inhibitor, NSC 23766 trihydrochloride (Cat: HY-15723A, Medchem express) (Fig. 7*H*). Furthermore, to ensure if in the absence/decreased ROCK1 level, Rac1 sustained its active state in enhancing phagocytosis, we performed another confirmatory experiment where BV2 cell were treated with various experimental treatment combination either alone or with Rac1 inhibitor followed by phagocytosis assay. It was observed that both in arsenic and ROCK1 siRNA treated group phagocytosis was increased significantly compared to control group, which was reversed by inhibition of Rac1 activity using Rac1 inhibitor, suggesting a role of active Rac1 downstream to ROCK1 in controlling microglial phagocytosis (Fig. 7*H*). Finally, to check the activity state of Rac1, BV2 cells were exposed to only arsenic and arsenic + anti-miR followed by pull-down of active Rac1 protein (Rac1-GTP) using Active Rac1 pull-down and Detection Kit (Cat: 16118, Thermo fisher) and Western blotting of pull-down sample was performed. An increased level of active Rac1 was detected in an arsenic-exposed group compared to control, which was reversed by anti-miR (Fig. 7*I*). However, no change was observed in the level of total Rac1 in the input sample (Fig. 7*J*). These observations further confirmed that arsenic exposure increases the level of active Rac1, while decreasing the level of ROCK1, which in turn enhances microglial phagocytosis. Therefore, it has been evident from the study that the phagocytic signal flows through the miR-129-5p→ROCK1→Rac1→phagocytosis axis.

## Discussion

Phagocytosis is the key function of microglia, which helps in synaptic pruning, clearing pathogens as well as cellular debri in the central nervous system in normal and various disease conditions like Alzheimer’s Disease (AD), Parkinson's Disease (PD), Multiple Sclerosis (MS) and others (1, 34). Therefore, the regulation of phagocytosis has huge clinical importance. In the present study, we unravel the underlying mechanism of miR-129-5p-mediated regulation of microglial phagocytosis by employing *in silico* analysis, *in vitro* experiments with the microglial cell line, BV2, primary microglia, as well as *ex vivo* microglia. The results were further validated in *in vivo* settings using BALB/c mice.

Microglial phagocytosis can be influenced by various pathophysiological conditions or environmental factors. Impaired phagocytosis has been reported in various neurodegenerative diseases such as AD, PD, and MS (1, 34). Recently, we showed that perinatal exposure to the environmental contaminant, arsenic, increases microglial phagocytosis (35, 36). In contrast, adult exposure for a shorter period has been shown to impair the phagocytic potential of microglia (37). Unlike microglia, peripheral blood monocytes isolated from arsenic-exposed individuals aged 15 to 65 years showed lower expression of phagocytic markers, Fcγ receptor, and complement receptors (38), as well as reduced phagocytosis of FITC-labeled *S. typhimurium* (39). Consistent with our earlier studies (35, 36), the present study also showed an increased phagocytosis not only by *ex vivo* microglia but also by primary neonatal microglia, BV2 mouse microglia cell line, and human microglia cell line CHME3.

ROCK1 is involved in the regulation of microglial activity (5). In the present study, arsenic has been shown to decrease the expression of ROCK1, and simultaneously, microglial phagocytosis was observed to be increased. Involvement of ROCK1 in microglial phagocytosis was confirmed by inhibiting ROCK1 using siRNA, which is well supported by the existing studies using J774 macrophage cell line (7) and microglia (22). In contrast, inhibition of Rho/Rock signaling by small molecule agents reported to compromise microglial phagocytosis of neuronal debri (40). Interestingly, the blockade of Rock has been found to enhance the phagocytosis of dendritic cells (8). Similarly, an increased phagocytosis of myoblast progenitor cells (mpcs)-apoptotic body by murine bone marrow-derived macrophage was observed following inhibition of RhoA-ROCK1 pathway (6).

A few earlier studies showed that the mRNA levels of target genes like Rbfox, Kv1.1, and EGFR did not change significantly under experimental conditions, but the protein levels were altered (41, 42, 43). A similar effect was observed in microglial ROCK1 expression following arsenic exposure in the present study, which aligns with our earlier study where miR-129-5p was shown to control TNF-α/IL-6 by targeting the microglial surface receptor, CD200R1 (32). The differential expression of mRNA and protein of any gene indicates post-transcriptional regulation, and miRNAs are prominent post-transcriptional regulators (44). Therefore, we focused on the role of miRNA in the regulation of ROCK1 following arsenic exposure. A TaqMan low-density array was run for global profiling of miRNA. A total of 15 significantly up-regulated (≥1.5-fold) and 18 significantly downregulated (≤0.8-fold) miRNAs were identified, and network analysis was performed using the Ingenuity Pathway Analysis (IPA) tool. The network analysis provided a preliminary idea of the relationship between interacting molecules such as arsenic, TNF, IL-6, CD200R1, DNMT1, CD200, ROCK1, cellular functions like phagocytosis, and dysregulated miRNAs.

MiRNAs control translation by binding to the 3′ UTR, coding region, or 5′ UTR of specific mRNAs (44, 45). *In silico* analysis (46) in the present study predicted two potential binding sites of miR-129-5p in the 3′ UTR of mouse ROCK1. The two predicted binding sites were validated by generating the luciferase reporter plasmid containing WT and mutant 3′ UTR sequences of ROCK1. The significant reversal of luciferase activity in the mutant 2 and mutant 3 (containing double mutations) plasmid-transfected cells proves that site 2 is involved in miRNA binding, whereas site 1 is not involved (32). A few miRNAs have been reported to regulate the expression of ROCK1 in various types of cancer, such as miR-124, miR-124-3p, miR-135a, miR-144, miR-148a, miR-150, miR-196a-5p, miR-584-3p, miR-1280, and miR-186 (15, 16, 17, 18, 20, 21, 22, 23, 24, 25). Besides cancer, miRNAs are also involved in normal physiological processes. For example, miR-145 and miR-148a have been reported to be involved in cell proliferation and myogenic differentiation (27, 29).

Once the miRNA binds to its target mRNA, it can either lead to mRNA degradation or be guided to the cytoplasmic processing body, p-body (32, 47), depending on the cellular requirement (48, 49, 50). In the present study, p-bodies were observed in both arsenic and pre-miR-treated microglia. The increased p-body formation following arsenic treatment indicates that the ROCK1 mRNA, upon binding to the miR-129-5p, goes to the p-body, which was again well supported by the protection of ROCK1 mRNA degradation in arsenic and pre-miR-treated cells following actinomycin-D treatment (51). A non-significant increase in the level of ROCK1 mRNA in pre-miR-129 and arsenic-treated groups compared to control, signifies a possible inclusion and storage of mRNA during the 72 h pre-incubation before the addition of Actinomycin D. An enrichment of miR-129-5p and ROCK1 mRNA in the p-body immuno-precipitated sample further confirms the inclusion of ROCK1 mRNA following binding to miR-129-5p, thereby inducing translational repression of ROCK1.

MiRNAs are also known to influence the phagocytosis of apoptotic cells, bacterial or protozoan pathogens and zymosan particles by macrophages of various origin by targeting specific proteins like WAS and VASP, CEBP-δ, CLTC1,PKCα, TRAF6, IRAK, ALX/FPR2, CD47, SIRPα, TIRAP (52). Therefore, our next obvious thought was to check whether miR-129-5p is involved in microglial phagocytosis or not. *In vitro* and *in vivo* experiments using arsenic, pre-miR and anti-miR proved that miR-129-5p is involved in the phagocytic activity of microglia. Similar to miR-129-5p in the present study, other miRNAs like miR-34, miR-98 and miR-155, miR-124 and miR-340 have also been reported to influence the phagocytosis of Aβ42, diseased neuron, myelin and latex beads respectively by controlling the expression of various target protein (30, 53, 54, 55). Interestingly, none of the miRNAs discussed above has been shown to influence phagocytosis by targeting ROCK1 in any cell types. Therefore, the association between microglial miR-129-5p and phagocytosis has been established in the present study. Finally, experiments were performed by using various combinations of arsenic, anti-miR and ROCK1 siRNA and it clearly showed that in microglia miR-129-5p controlled phagocytosis through regulation of ROCK1 (32).

Rac1, a downstream target for ROCK1, plays a crucial role in membrane ruffling and phagocytosis through regulation of cytoskeleton dynamics (9, 10, 33). Interestingly, a negative feedback loop exists between RhoA, ROCK1 and Rac1, where ROCK1 negatively regulates Rac1 activity and active Rac1 inhibits RhoA, while activated RhoA positively regulates ROCK1 (33). However, the connection between ROCK1, Rac1, and Phagocytosis is not well-known. In this study, we observed Rac1 siRNA and Rac1 activity inhibitor significantly reversed arsenic-induced increased phagocytosis by BV2 microglia, despite no effect of arsenic was noted on the expression of Rac1. Similarly, Rac1 inhibitor also reversed increased phagocytosis in ROCK1 knockdown BV2 cells. These data strongly support that a decrease in ROCK1 protein level enhances phagocytosis *via* sustaining Rac1 in its active GTP-bound state in arsenic-exposed microglia, which was strongly proven by detecting the increased level of active (GTP-bound) Rac1 in arsenic-exposed microglia and its reversal following anti-miR-129-5p co-exposure with arsenic.

To further ensure the sustained active state of Rac1, another experiment was performed where the active RhoA-GTP level was evaluated using the Active Rho Detection Kit. A significant decrease in the level of active RhoA was observed in arsenic-exposed BV2 cells compared to control and was restored by Anti-miR treatment (Fig. S3). This data indicates that decreased ROCK1 protein activates its downstream molecule Rac1 (Rac1-GTP) in arsenic-exposed BV2 cells, which in turn inhibits RhoA activation. While treatment with anti-miR increased the level of ROCK1 protein in arsenic-exposed BV2 cells, which in turn restored RhoA activity by inhibiting Rac1 activity. Collectively, our findings comprehensively established that the phagocytic signal flows through the miR-129-5p→ROCK1→Rac1→phagocytosis axis.

**Figure 8:**
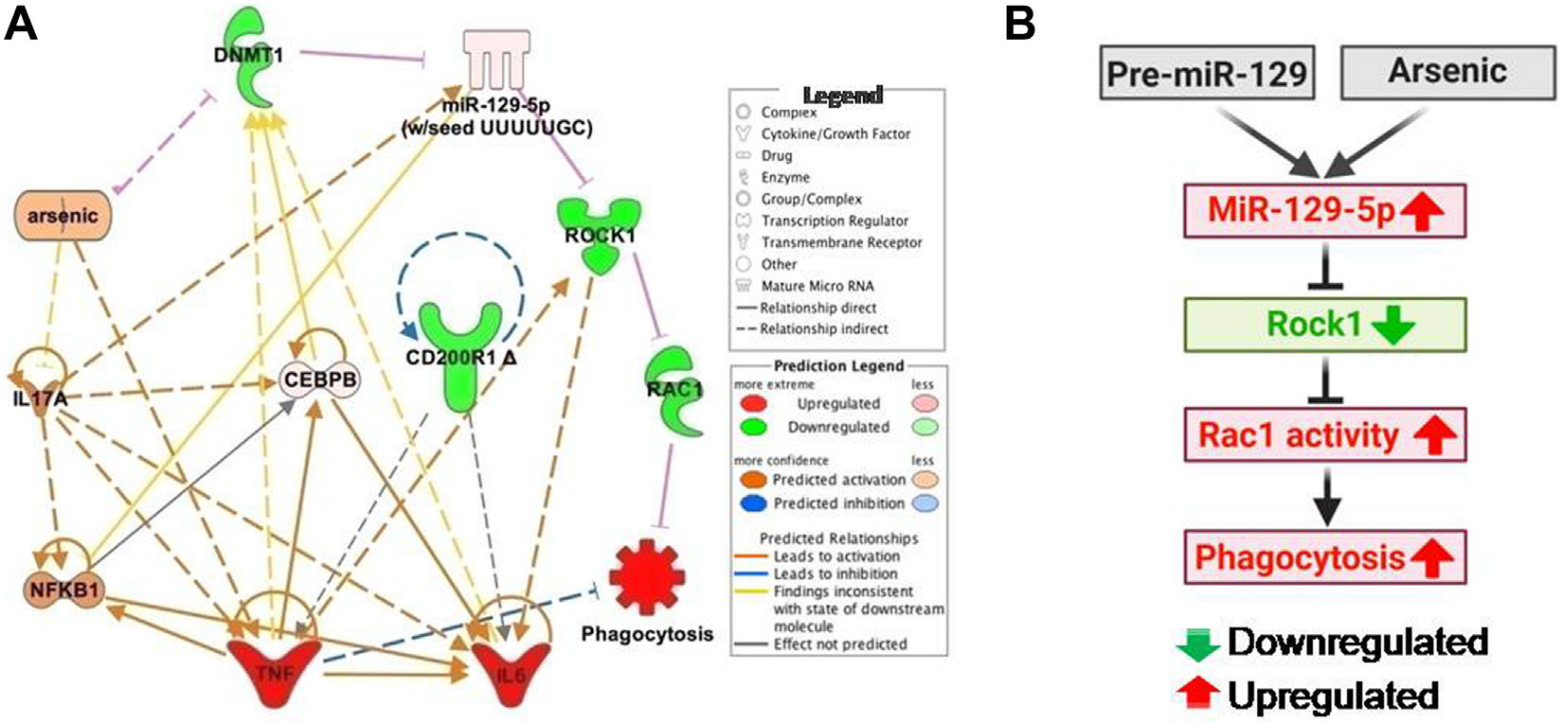
**Regulation of microglial phagocytosis by miR-129-5p.***A*, plausible relationship between arsenic, DNMT1, miR-129-5p, ROCK1 and phagocytosis deduced from the results of the study is shown here in an IPA generated network with the new link shown in *pink*. The symbols are indicated in the legend. Significantly downregulated genes are in *green*, upregulated are in *red*. *B*, proposed signaling axis of miR-129-5p-mediated regulation of microglial phagocytosis.

Overall, we propose miR-129-5p as a key epigenetic factor that controls the phagocytic function of microglia through post-transcriptional regulation of ROCK1. Arsenic increases the expression of miR-129-5p by inducing demethylation of the CpG islands in its promoter region (32). MiR-129-5p binds to the 3′ UTR of ROCK1, thereby inducing its translational repression. Decreased level of ROCK1 in turn activates its downstream molecule Rac1, which, finally, leads to the increased microglial phagocytosis (Fig. 8, *A* and *B*). Therefore, controlling miR-129-5p, an important regulator of microglial phagocytosis, represents a potential strategy for therapeutic intervention of microglial phagocytosis in altered synaptic plasticity as well as various neurodegenerative diseases like Alzheimer’s disease, Parkinson’s disease, Multiple Sclerosis, and others.

## Experimental procedures

### Reagents and antibodies

Sodium arsenite (SA) (NaAsO2, 90,110 K02), Percoll (P4937–500 Ml), cell culture medium (DMEM/F12, 12500096), Papain, sodium dodecyl sulfate (SDS, L3771–100 G), 30% Acrylamide/Bis Solution (1610156), bromophenol blue (B5525–10 G), Tween 20 (SL9S690890), Triton-X 100 (T8787–250 ml), EDTA, protease inhibitor cocktail (P8340–1 ml), methanol (LC grade) and PBS (D5652–50L) were obtained from Sigma. PVDF membrane (IPVH00010) and chemiluminescence substrates (WBKL50500) were obtained from Merck-Millipore. The Rac1(4651T, CST, dilution 1:2500) PSD95 (3450S, CST, dilution 1:1000), Iba1 (MABN92, Millipore, dilution 1:1000), and ROCK1 antibody for Western blot analysis & immunohistochemistry were procured from Cell signaling technology (4035S, dilution 1:2500) and G-biosciences (ITT4162–100u, dilution 1:1000) respectively. Beta Actin (sc-47778), GAPDH (sc-47724), and GW182 (for WB sc-377006, for IP sc-56314) were obtained from Santa Cruz. Rac1 inhibitor, NSC 23766 trihydrochloride (HY-15723A), was obtained from MedchemExpress. Fetal Bovine Serum (FBS, Gibco, A4766801), Cell culture medium DMEM/F12 (12500096) and MEM (61100-103), TaqMan Array Rodent MicroRNA A+B Cards Set v3.0, Megaplex RT Primers, Megaplex PreAmp Primers, TaqMan Universal PCR Master Mix (2X, 4440043), mirVana miRNA Isolation Kit (AM1560), miR-129-5p (4427975 000590, Assay ID: 000590) & U6 (4440887, Assay ID: 001973) TaqMan real-time assay kit, pre-miR-129 (assay ID: PM10195, ABI), anti-miR-129-5p (assay ID: AM10195, oligo sequence: 5′GCAAGCCCAGACCGCAAAAAG3′), ROCK1 siRNA (4390771, Assay ID: s203580), HRP-anti-mouse (A -11029), HRP-anti-rabbit (656120), Alexa Fluor 594 anti-rabbit (A-11037), Alexa Fluor 488 anti-mouse (A11029), DNA co-immunoprecipitation kit (10007-D), CellTracker CM-DiI Dye (C7001), and Trypan Blue Solution (0.4%, 15250061) and cDNA synthesis kit (4368814) were obtained from Thermo Scientific. Tissue freezing medium for cryosectioning was obtained from Leica. Plasmids containing the 3′UTR of ROCK1 (WT and mutants) for luciferase activity were outsourced to Thermo Scientific. Primers for qRT-PCR were obtained from Integrated DNA Technology (IDT). The primers sequences were as follows, β-Actin (forward-5′-TATGGAATCCTGTGGCATC′-3′; reverse-5′-GTGTTGGCATAGAGGTCTT′-3), ROCK1 (forward-5′-AACATGCTGCTGGATAAATCTGG-3′; reverse-5′-TGTATCACATCGTACCATGCCT-3′), Rac1 (forward-5′-CCAGATGCAGGCCATCAAGT-3′; reverse-5′-GCAGGCAGGTTTTACCAACAG-3′). Protein ladder (PG-PMT2922) was obtained from Puregene. Tris/glycin/SDA running buffer (1,610,772), Tris/glycin transfer buffer (1,610,771) were procured from Biorad. The siRNAs for ROCK1 (siRNA ID: s203580) and Rac1 (siRNA ID: s72647) were procured from Thermo Fisher Scientific. The sequence of ROCK1 siRNA is 5′->3′ Sense-GGUUGGGACUUACAGUAAAtt and Antisence- UUUACUGUAAGUCCCAACCaa. The sequence of Rac1 siRNA is 5′->3′ Sense-CAGACAGACGUGUUCUUAAtt and Antisence-UUAAGAACACGUCUGUCUGcg.

### Animal husbandry and treatment

Six- to eight-week-old male and female BALB/c mice were procured from the animal breeding facility of the CSIR-Indian Institute of Toxicology Research (CSIR-IITR). Protocols used in the study were approved by the Institutional Animal Ethics Committee (IAEC) of CSIR-IITR (IAEC-IITR). We have performed all animal experiments following the guidelines laid down by the committee for the purpose of control and supervision of experiments on animals (CPCSEA) and the Ministry of Environment and Forests (MoEF), Government of India, New Delhi, India. The IAEC reference no is IITR/IAEC/31/22. Mice were brought from the breeding facility to the experimental facility and housed at 25 ± 3 °C, 12 h/12 h light and dark cycle with rodent chow diet (Cat. no: 1324, Altromin, Germany) and reverse osmosis (RO) water supplied ad libitum. After 10 days of acclimatization animals were used for the experiment. For mating, male and female animals were kept in the same cages in a ratio of 1:2 (male:female)/cage. Mating was confirmed by checking the formation of vaginal plugs in female mice. After timed mating, pregnant females were randomly assigned to either the vehicle control or arsenic-treatment groups (0.38 mg/kg bd wt). Sodium arsenite (arsenic) was dissolved in RO water and the pregnant females of the treatment group were gavaged daily with arsenic and the females of the vehicle control group received only RO water. The treatment regimen continued from gestational day 5 (GD5) until the weaning of pups around post-natal day 22 (PND22). At the age of PND17, some pups from both control and arsenic treated group were underwent stereotactic surgery to inject anti-miR-129-5p into their lateral ventrical of brain which created two new additional experimental groups designated as “anti-miR” and “arsenic + anti-miR”. Following the exposure regimen, pups were sacrificed for collection of brains. Before collecting the brain tissues, animals were euthanized by overdosing of ketamine: xylazine, the hippocampus were isolated from the pups brain and processed either for Western blot analysis or quantitative real-time PCR (qRT-PCR). For the isolation of microglia (*ex vivo* microglia), whole brain was used. For the purpose of immunohistochemical staining, after deep anesthesia the mouse was perfused first with 0.01 M PBS followed by 4% PFA in 0.01 M PBS through the left cardiac ventricle and processed further as described in immunohistochemistry (IHC) section.

### Isolation of primary microglia and treatment

#### Isolation of primary neonatal microglia and treatment

Neonatal microglia were isolated from postnatal day 01 to 03 old pups following the protocol published from our lab earlier (56). Briefly, brains were dissected out and homogenized using a 5 ml syringe to form mixed glial cell suspension. Mixed glial cells (0.4 × 10^6^) were seeded in 12-well plates in DMEM/F12 medium supplemented with 10% FBS and 1% pen-strep followed by media replacement after 48 h. The culture medium was replaced every fourth day until full confluency at around 30 days. Mix glial culture was incubated for 30 to 45 min in serum-free media and trypsin solution (1:1 ratio), followed by the removal of floating cells. The attached cells are pure microglia, and these cells were used for further experiments.

#### Acute isolation of primary microglia from adult mouse brain

Primary adult microglia (*ex vivo* microglia) were isolated (acute isolation) following the protocol published earlier from our lab (37). Briefly, brain samples were chopped and enzymatically digested with papain (20 U/ml) at 37 °C for 20 min. The resulting digested tissue was mixed with 30% isotonic Percoll and centrifuged at 500*g* for 20 min at 20 °C. Isolated cells were immediately processed for RNA isolation using the mirVana kit or cultured at a density of 5 × 10^4^ cells/well in a 96-well culture plate for phagocytosis assay.

### Cell line maintenance and treatment

Mouse microglia cell line (BV2) and human microglia cell line (CHME3) were cultured in complete DMEM/F12 medium supplemented with 1% penicillin-streptomycin (Pen-Strep) and 10% fetal bovine serum (FBS) in a CO_2_ incubator maintaining 37 °C temperature, 5% CO_2,_ and 95% humidity. Cells were passaged when they reached 80 to 90% confluency. HEK293 cells were cultured in DMEM medium supplemented with 1% penicillin-streptomycin (Pen-Strep) and 10% Fetal Bovine Serum (FBS).

#### *In vitro* treatment

For phagocytosis assay BV2 and CHME3 cells were seeded @ 2500 cells/300 μl/well of 96-well culture plate (black or transparent) and 8000 cells /300 μl/well of 96-well culture plate (black), respectively. Cells were treated with a non-cytotoxic dose of arsenic (1 μM) for 72 h and other reagents as required. For isolation of protein for Western blot analysis and RNA for PCR analysis, BV2 and CHME3 cells were seeded in 6-well culture plate @ 60,000/3.5 ml/well/6 well culture plate and 250,000/3.5 ml/well/6-well culture plate, respectively, and incubated for 72 h with arsenic (1 μM). Rac1 inhibitor was used to investigate the role of active Rac1in the regulation of phagocytosis. For this experiment, BV2 cells were treated with 10 μM Rac1 inhibitor 6 h prior to the completion of the 72 h incubation.

For luciferase assay, HEK-293 cells were seeded @ 8000 cells/300 μl/well of 96-well culture plates. Cells were transfected with various combinations of WT & mutant 3′ UTR of ROCK1 and pre-miR-129-5p, incubated for 72 h followed by checking luciferase activity.

### Preparation of neuronal debri for phagocytosis assay

To conduct a phagocytosis assay, neuronal debri stained with CellTracker CM-DiI dye (C7001, Thermo Fisher scientific) was generated, which mimics the cellular debris in the CNS (57). Neuro-2a (N2a) cells were used to generate neuronal debri. N2a cells were seeded @ 300,000 cells/T-25 flask in complete DMEM-F12 medium and allowed to grow till 80 to 90% confluency was achieved. The culture medium was aspirated, and the cells were rinsed with sterile PBS. A working solution (2.5 nM) of the CM-Dil dye was freshly prepared by adding 5 μl of stock dye solution (1 μM) in 2 ml of DMEM-F12 medium without any supplement, added to the N2a culture and incubated for 2 h incubation in dark condition. Following incubation, the culture medium containing CM-Dill dye was removed, washed with PBS, and 2 ml of fresh DMEM-F12 medium added. To induce apoptosis of stained N2a cells, 10 μl H_2_O_2_ (stock concentration 1 mM) was added to the flask to make a final concentration of 5 μM and incubated for 24 h in a CO_2_ incubator (57). Following incubation, the culture medium containing the CM-DiI-stained apoptotic bodies was collected and centrifuged at 4000*g* for 10 min. The supernatant was removed and pellet was rinsed with sterile PBS at 4000*g* for 10 min. Finally, apoptotic bodies were resuspended in 500 μl of sterile PBS. These apoptotic bodies are called as “neuronal debri”. A bulk amount of (∼15 ml) neuronal debri was generated in a single batch and was used in all the experiments in the present study. These debri should be protected from light and can be stored in −20 °C for long-term use.

### Phagocytosis assay

To investigate the alteration of microglial phagocytosis in various treatment conditions, BV2, CHME3, primary neonatal microglia, and *ex vivo* microglia isolated from PND22 mouse pups were used. Different microglial cell systems were used to check the commonality of the response from microglia of different origins. BV2, CHME3, and primary neonatal microglia were treated either with arsenic, pre-miR, or anti-miR in a CO_2_ incubator for 72 h as described in the cell line and treatment section. CM-DiI dye-stained apoptotic neuronal debri (5 μl/300 μl medium/well of 96-well culture plate) was added 3 h before the endpoint of 72 h incubation and allowed for phagocytosis by microglia. For *ex vivo* microglia, cells were isolated from the brain of animals of various treatment groups *viz.* Control, arsenic-treatment, and arsenic + anti-miR-treatment groups and seeded @100,000 cells/well in a 96-well black culture plate in complete culture medium. After 30-min incubation, the media was replaced with 300 μl DMEM-F12 containing CM-DiI-stained apoptotic neuronal debris (5 μl) and incubated for 3 h.

Two different methods were used to measure the phagocytic activity. The first method was based on imaging of microglia with neuronal debri. Cells were incubated with neuronal debri as described earlier for 3 h. After incubation, neuronal debri containing media was aspirated and cells were fixed with 4% PFA for 15 to 20 min. Cells were washed four times as described previously with PBS. Finally, images were captured using a fluorescence microscope (EVOS 5000, Thermo). Neuronal debri-associated fluorescence in each microglia was analyzed using ImageJ software (Fiji) and expressed as neuronal debri-associated fluorescence/cell. The second method was to measure the fluorescence of phagocytosed neuronal debri in a micrplate reader. Cells were incubated with neuronal debri as described earlier for 3h. After incubation, neuronal debri containing media was aspirated and cells were washed with 1X PBS for 5 min thrice on a rocker to wash out reminiscent debris those were not phagocytosed by microglia. Further to mask the fluorescent signal of the debri adhered to the cell membrane, 0.1X trypan blue solution was added (100 ul/well) for 1 to 2 min and then removed. Cells were further washed twice as mentioned earlier in this section. Finally, 150 μl of PBS/well was added, and mean fluorescent intensity was measured using a plate reader (Cytation|5, Biotek) (35). The fluorescence was expressed as Neuronal debri-associated fluorescence/well of 96-well culture plate.

### Preparation of cell/tissue lysate and Western blot analysis

Preparation of cell lysate or brain tissue lysate (hippocampus) and subsequent Western blot analysis was performed following the protocol published earlier from our laboratory (32) In brief, cells in 6 well culture plates were washed in cold PBS and scrapped in 45 μl of cell lysis buffer (20 mM Tris–HCl pH-8, 137 mM NaCl, 10% glycerol, 1% Triton X-100, 2 mM EDTA, and protease inhibitor cocktail). Lysed cells from each well were collected in different 1.5 ml tubes and vortexed for 10 s every 3 min × 5 times. For the preparation of tissue lysate, the hippocampus was isolated from the PND22 mouse brains and homogenized in the lysis buffer. Finally, the cell/tissue lysate was centrifuged at 14,000*g* for 15 min at 4 °C, and the supernatant collected and used for Western blot analysis. Protein concentration was estimated by BCA Kit (23235, Thermo). An equal amount (20–25 μg) of protein of each sample were run on 10% SDS-PAGE, transferred to PVDF membrane, and probed with the desired primary antibody diluted in TBS with tween-20 (0.05%) followed by incubation with HRP-tagged secondary antibody. Blots were observed in a gel documentation system (G-box H-16, Syngene and Amersham Imager 600, GE Healthcare) using Immobilon substrate (Millipore, WBKL50500). Images of the target protein bands and bands of endogenous control protein (GAPDH or β actin) were captured and used for densitomentric analysis. Densitometric analysis was performed by using Image-J software (NIH, https://imagej.nih.gov/ij/). The value of target protein band intensity was normalized against the band intensity of the respective endogenous control and expressed as fold change over control group.

### Taqman low-density array (TLDA) for miRNA

TaqMan Low Density Array (TLDA) for miRNA was run following the published protocol from our lab (32). The expression of 641 miRNAs was studied by using TaqMan Low-Density Arrays Pool A and pool B cards (TaqMan Rodent MicroRNA Set Cards v3.0: Part no. 4398979 for pool A contains 317 and part no. 4455449 for pool B contains 324 miRNA primers) from Thermo Scientific were used. In brief, the total RNA was isolated from *in vitro* arsenic-treated (72 h) primary neonatal microglia using mirVana kit. RNA (300 ng) was subjected to reverse transcription (RT) by using megaplex RT primers of pool A and pool B. After reverse transcription, preamplification was carried out using TaqMan preamp master mix and megaplex preamp primers of pool A (part no. 4399203) and pool B (part no. 4444308). Finally, for a single plate, 450 μl of TaqMan universal PCR master mix was mixed with 9 μl of preamp product along with 441 μl nuclease-free water. In each port of the TLDA plates, 100 μl from the above-mentioned master mixture was added, and after sealing and spinning, the plates were loaded on a QuantStudio 12K Flex Real-Time PCR system (Thermo). Relative quantification was done using ^-ΔΔ^Ct method, considering the levels of endogenous controls with the expression suite online software (Thermo).

### Real-time PCR of miRNAs

The total mRNA was isolated using the mirVana kit from control and treated cells, as well as hippocampus tissue. The level of miR-129-5p was detected using specific TaqMan microRNA assays (part number 4373068, Applied Biosystems) and TaqMan Universal PCR Master Mix, No AmpErase UNG (part number 4324018; Applied Biosystems) following the manufacturer’s instructions in aquantstudio 6 Flex real-time PCR system. The level of miRNA expression was measured by relative quantification performed using ^∼ΔΔ^Ct method, where Ct values of miR-129-5p were normalized to the Ct value of U6 from the same sample for each group.

### Real-time PCR

Total RNA was isolated either from hippocampus tissue or cell pellets using Trizol reagent (Invitrogen). The concentration of total RNA was determined using a Nanodrop spectrophotometer (Thermo). cDNA was synthesized using a high-capacity cDNA reverse transcription kit (4368814, Thermo). The qRT-PCR was run in a QuantStudio 6 flex real-time PCR system (Thermo) using SYBR green master mix (PGK022-B, Puregene) and specific forward–reverse primers. Mouse β-actin was used for relative quantification following ^∼ΔΔ^Ct method.

### Immunohistochemistry and analysis of colocalization

For immunohistochemistry, animals were deeply anaesthetized with ketamine and xylazine injection (60:20 mg/kg bd wt) at PND22 and perfused with 0.01 M PBS followed by 4% PFA in 0.01 M PBS through the left cardiac ventricle. The whole brains were dissected out and fixed in 4% PFA overnight at 4 °C. After PFA fixation, the whole brains were transferred to 15% and 30% sucrose solution (prepared in 0.01 M PBS) and kept overnight in each gradient at 4 °C. Frozen tissue blocks were prepared using Cryomatrix at −20 °C, and 14 μM coronal cryo-sections were cut (−1.8 mm to −2.3 mm from bregma, hippocampus region) in a cryo-microtome (Microm HM 520, Labcon, Germany). Tissue sections were collected in PBS as free-floating sections and stored at 4 °C if not used immediately (not more than a week). Free-floating sections were washed with 0.1% PBST (0.1%TritonX-100 in PBS) for 3 × 10 min. For antigen retrieval, tissue sections were submerged in 0.01 M sodium citrate buffer and kept in a microwave oven till the buffer started boiling. Thereafter, tissue sections were permeabilized with 1% PBST for 60 min at room temperature. The permeabilized tissue sections were incubated for 1 h at RT in blocking buffer (0.1% TritonX-100 and 10% horse serum in PBS) followed by probing with primary antibodies diluted in blocking buffer at 4 °C for 48 h. Following incubation with primary antibodies, sections were again washed with 0.1% PBST 3 × 10 min and incubated with AlexaFluor-conjugated diluted (1:500) secondary antibodies for 2 h in the dark at RT. Finally, sections were counterstained with DAPI-containing mounting medium and left it for drying in the dark. Slides were observed and images were captured in a confocal microscope (Stellaris|8, Leica). For analysis of colocalization of Iba1 and PSD95, fluorescent-merged images were split into its 3 RGB components (8 bit grayscale) using the image/color/RGB split function of ImageJ (NIH, https://imagej.nih.gov/ij/). These components were subsequently imported into the JACoP plugin (just AnotherCo-localization Plugin of imageJ) for thresholding and calculation of the coefficient of red and green colocalization (Manders' Coefficients).

### P-body immunostaining

Microglia were treated either with arsenic or with pre-miR-129 and cultured on a coverslip in a 12-well culture plate for 72 h. After treatment, cells were washed with PBS and fixed in 4% paraformaldehyde, followed by incubation with blocking buffer (1 PBS + 2% FBS+0.05% Tween-20) for 1 h at room temperature. GW182 primary antibody was added to the cells and incubated overnight at 4 °C. Cells were washed 3 × 10 min and further incubated with fluorescence-tagged secondary antibody for 2 h at room temperature. Finally, coverslips were mounted on a glass slide with DAPI-containing antifade mounting medium (Vector Lab, H-1200) and observed under a fluorescence microscope for proper staining. Images were captured in a confocal microscope (Zeiss, Germany), and the number of p-body (GW182-associated fluorescent dots) was counted.

### Transfection of ROCK1 siRNA, Rac1 siRNA, pre-miR-129-5p, anti-miR-129-5p and GW182 plasmid

Microglia (60,000 cells/well in a 12-well culture plate) were transfected with ROCK1 siRNA (30 nM) (Fig. S1), Rac1 siRNA (30 nM) (Fig. S2), pre-miR-129 (10 nM) and anti-miR-129-5p (100 nM) alone or in combination using siPORT NeoFX transfection agent. Briefly, transfection reagent was mixed with opti-MEM, followed by the addition of ROCK1 siRNA, pre-miR-129-5p or anti-miR-129-5p kept at room temperature for 10 min 100 μl resulting transfection complex was added to microglia for 72 h and processed for Western blot analysis or qRT-PCR. For the transfection of GW182 plasmid, cells were transfected using FuGENE Transfection Reagent (E2691, Promega). Fugene was mixed with Opti MEM, and the mixture was incubated for 5 min. Then, plasmid (3 μg) was added into the mixture, incubated for 15 min, and the transfection complex was added into the cells in a T75 flask. Transfection efficiency was checked by Western blot analysis.

### Bioinformatic analysis

Bioinformatic analysis was performed as described in published article from our lab (32). Significant fold changes of miRNAs calculated from ExpressionSuite software (Thermo) were visualized as a heat map, and the cluster was generated with Cluster 3.0 software (http://bonsai.hgc.jp/∼mdehoon/software/cluster/software.htm). Fold changes of the miRNAs were then submitted to Ingenuity Pathway Analysis (IPA, QIAGEN Inc. https://www.qiagenbioinformatics.com/products/ingenuity-pathway-analysis) for core analysis based on experimentally observed and predicted data resourced from the Ingenuity Knowledge Base. The top canonical pathways, diseases, and functions, and gene networks that are most significant to microarray studies were identified, and differentially expressed genes in specific diseases and functions were categorized. These genes were then used to generate functional networks between miRNAs and their target molecules. For miRNA target prediction, initially, TargetScan (http://www.targetscan.org/mmu72/) was used, followed by RNA22 (https://cm.jefferson.edu/rna22/) and RNA hybrid (https://bibiserv.cebitec.uni-bielefeld. de/rnahybrid/) software for obtaining free energy and structure.

### 3′-UTR luciferase reporter assay

Wild-type (WT) and mutant (Mut1, Mut2, and double mutant) plasmids of 3′UTR of ROCK1 mRNA were custom-designed from Thermo (Thermo Fisher Scientific). Wild-type and mutant 3′UTR were sub-cloned into the MluI and SpeI sites of the pMIR-report vector. These products are referred to as pMIR-ROCK1-WT for wild-type and pMIR-ROCK1-Mut 1, pMIR-ROCK1-Mut 2 & pMIR-ROCK1-Mut 3 (double mutant) for the wild-type and mutant 3′UTR from the ROCK1 gene, respectively. HEK-293 cells (5000 cells/well) were seeded at in a 96-well tissue culture plate along with siPORT NeoFX transfection agent containing pre-miR-129 for 8 h. After transfection, the medium was removed, and cells were rinsed, followed by transfection with pMIR-reporter, pMIR-ROCK1-WT, pMIR-ROCK1-Mut 1, pMIR-ROCK1-Mut 2 & pMIR-ROCK1-Mut 3 (double mutant) and pMIR-galactosidase vectors using Fugene transfection reagent. After 48 h of transfection, cells were harvested and lysed in 200 μl of lysis buffer, and the lysates were assayed for luciferase and galactosidase activity following standard protocols.

### mRNA stability assay

In order to check ROCK1 mRNA stability, microglia (60,000 cells/well of 12-well culture plates) were treated with arsenic (1 μM), pre-miR-129 (10 nM) for 72 h followed by the addition of actinomycin D (ActD; 1.5 μg/ml) to halt transcription. At 7 h post-ActD addition, cells were harvested and RNA isolated. Subsequently, ROCK1 mRNA levels were determined using qRT-PCR described earlier in the Materials and Methods section.

### RNA immunoprecipitation

For RNA immunoprecipitation, GW182 was over-expressed in BV2 cells by transfecting pmyc-GFP-TNRC6A plasmid (3 μg) for 24 h followed by treatment of arsenic (1 μM) for another 72 h. BV2 cells (1 × 10^6^) were seeded in T75 flasks and treated as required. After completion of the treatment, cells were fixed with 0.75% paraformaldehyde for 5 min, followed by neutralization in 125 mM glycine for an additional 5 min. Cells were scraped and lysed on ice. We have started the immunoprecipitation with 800 μg cell lysate and followed the protocol supplied with the Dynabeads CO-IP kit (Thermo). Finally, the immunoprecipitated pellet was resuspended in nuclease-free water and used to measure the level of ROCK1 mRNA and miR-129-5p RNA qRT-PCR.

### Intracerbroventricular injection of anti-miR-129-5p

Intracerbroventricular injection of anti-miR-129-5p was performed following published protocol from our laboratory (32). In order to confirm the role of miR-129-5p in regulating the expression of ROCK1 and phagocytosis, anti-miR-129-5p was introduced into the brain by intracerbroventricular injection using the stereotactic technique. Briefly, intraperitoneal injection of ketamine and xylazine (60 and 20 mg/kg body weight respectively) was given to deeply anesthetize each mouse pup before shaving its head and positioning it in the stereotactic frames (RWD Life Science). On the dorsal side of the head a midline scalp incision was performed in order to expose the skull and visualize bregma and lambda. Anti-miR-129-5p (0.25 nmole in 2 μl/brain, 1 μl in each hemisphere) was injected in each cortical hemisphere at the rate of (0.5 μl/min) into the cerebral cortex using a 10 μl Hamilton syringe at the following coordinates: 0.6 mm posterior, 1.5 mm lateral, and 1.3 mm dorsal with respect to bregma, once at PND17 and dissected at PND22 (58). The sham control group was treated with sterile nuclease free water that followed the same procedure. The microglia were isolated from animals of control and treatment group followed by phagocytosis assay. At the same time, hippocampus from the brain of experimental animals were isolated and processed for colocalization of Iba1 and PSD95.

### Active Rac1 pull-down and detection

Detection of active Rac1 (Rac1-GTP) was carried out using a GST-Pak1-PBD based pull-down and detection kit (Thermo Scientific, Cat. No.: 16,118). Detection of active Rac1 was performed following the manufacturer’s instructions. Briefly, BV2 cells were seeded (0.5 x 10^6^ cells/T25 flask) and treated with arsenic alone and in combination with anti-miR followed by 72 h incubation. After incubation, cells were lysed with Lysis/Binding/Wash Buffer and protein concentration were measured using Bradford reagent. A total of 800 μg protein was used for the assay from each group (Control, arsenic-treated and arsenic + anti-miR-treated). For the affinity precipitation of activated Rac1, protein lysate was incubated with GST-Pak1-PBD (20 μg) for 90 min at 4 °C using a column containing glutathione-resin agarose beads. The active Rac1 was eluted from the glutathione-resin agarose beads-based column using 40 μl reducing sample buffer from each control and treatment group. Finally, equal volume of eluted samples was run for Western blot analysis and the level of active Rac1 was detected by probing with Rac1 antibody. Fraction of the input samples were also run for Western blot analysis and the level of total Rac1 were also detected in each control and treatment group.

### Statistical analysis

GraphPad Prizm was used for making graphs and statistical analysis. All the data were presented as Mean ± SD (standard deviation) with individual data point as hybrid scatter-bar graph. To determine the homogeneity of variance “Normality test” was performed employing “Shapiro-Wilk normality test”. For comparing control and arsenic-exposed group Mann–Whitney test was performed and “One-way ANOVA” was used for comparing more than two groups followed by *post hoc* analysis by “Tukey’s test”. *p* < 0.05 was considered as statistically significant.

## Data availability

All data generated for this study are contained within the manuscript. For further queries corresponding author D. G. may be contacted.

## Supporting information

This article contains supporting information.

## Conflict of interest

The authors declare that they have no conflicts of interest with the contents of this article.
