# Supplementary material for: MicroRNA-129-5p-mediated translational repression of microglial ROCK1 leads to enhanced phagocytosis
Source: J Biol Chem. 2025 May 24;301(7):110293. doi: 10.1016/j.jbc.2025.110293 (PMC12221357; doi:10.1016/j.jbc.2025.110293)
Supplement: Supporting information [file mmc1.pdf]

MicroRNA-129-5p-mediated translational repression of microglial ROCK1 leads to enhanced phagocytosis

Running title: **MiR-129-5p and ROCK1 in microglial phagocytosis**

Rajib Kumar Dey[1,2], Ranjana Kumari[1,2], Roni Patra[1,2], Dharmendra Kumar Soni[3], Roopa Biswas[3], Satyakam Patnaik[4], Debabrata Ghosh[1,2]*

[1] Immunotoxicology Laboratory, Systems Toxicology Group, FEST Division, CSIR-Indian Institute of Toxicology Research (CSIR-IITR), Vishvigyan Bhawan, 31, Mahatma Gandhi Marg, Lucknow, Uttar Pradesh 226001, India.

[2] Academy of Scientific and Innovative Research (AcSIR), Ghaziabad 201002, India.

[3] Department of Anatomy, Physiology and Genetics, School of Medicine, Uniformed Services University of the Health Sciences, Bethesda, MD, 20814, USA

[4] Drug and Chemical Toxicology Group, FEST Division, CSIR-Indian Institute of Toxicology Research, Lucknow, Uttar Pradesh 226001, India

Address for correspondence:

*Debabrata Ghosh

Email: Debabrata.Ghosh@iitr.res.in; debabrataghosh78@gmail.com
ORCID: 0000-0002-6571-304X

Supporting information:

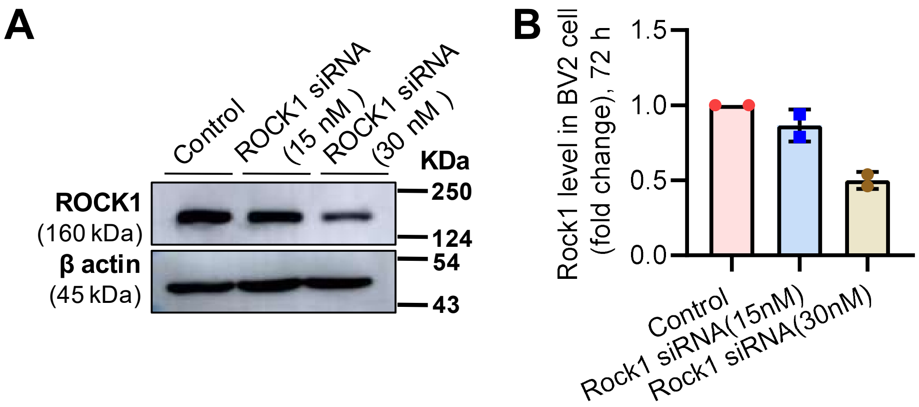

Figure S1. Dose determination of ROCK1 siRNA. BV2 microglia were transfected with ROCK1 siRNA (15 nM and 30 nM) using siPORT transfection reagent and incubated for 72 h. Following incubation, cells were harvested, protein isolated, run SDS-PAGE, transferred to PVDF membrane, probed with ROCK1antibody and visualized in a ChemiDoc instrument. The band intensities were calculated using ImageJ and presented as hybrid-scatter bar graph. The level of ROCK1 protein was observed to be lower in 30 nM siRNA treated group and it was used for further experiments. (A) Representative image of western blots, (B) Quantitative presentation of the band intensities of the western blots. N=2 independent experiments.

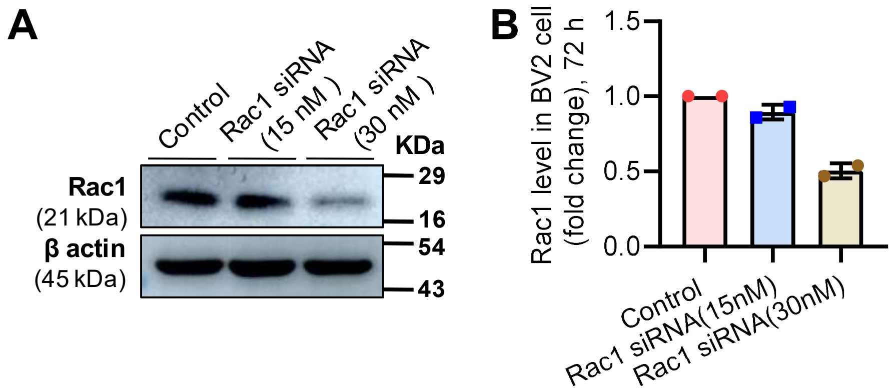

Figure S2. Dose determination of Rac1 siRNA. BV2 microglia were transfected with Rac1 siRNA (15 nM and 30 nM) using siPORT transfection reagent and incubated for 72 h. Following incubation, cells were harvested, protein isolated, run SDS-PAGE, transferred to PVDF membrane, probed with Rac1 antibody and visualized in a ChemiDoc instrument. The band intensities were calculated using ImageJ and presented as hybrid-scatter bar graph. The level of Rac1 protein was observed to be lower in 30 nM siRNA treated group and it was used for further experiments. (**A**) Representative image of western blots, (**B**) Quantitative presentation of the band intensities of the western blots. N=2 independent experiments.

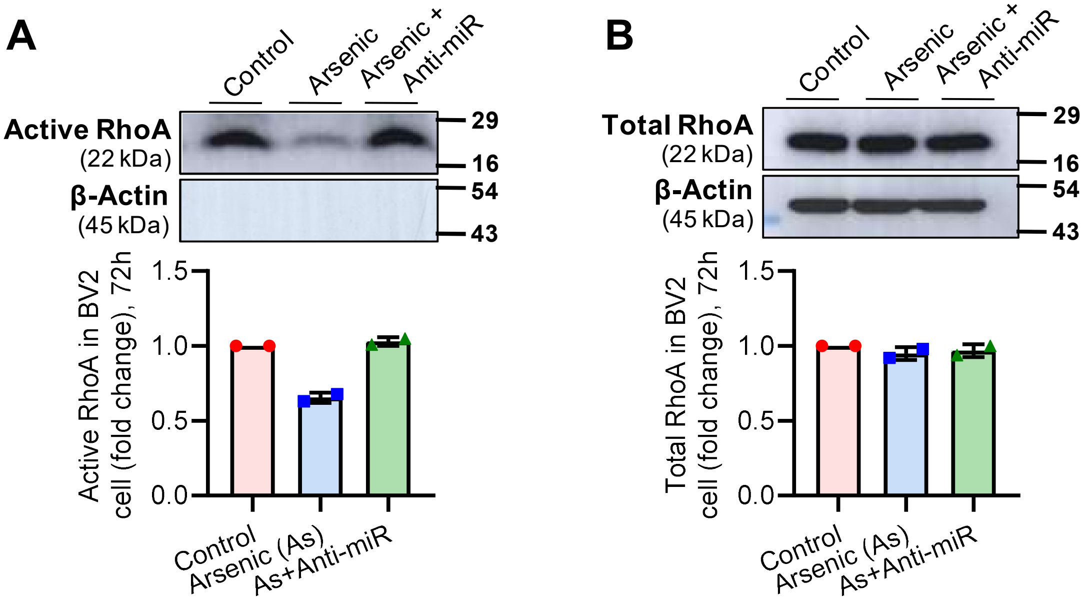

Figure S3. Effect of arsenic and anti-miR-129-5p on the level of active RhoA. BV2 microglia were exposed to arsenic and anti-miR-129-5p for 72h *in vitro* and processed for detection of Active RhoA using Active Rho Detection Kit (Cat: 8820, Cell Signaling Technology). The manufacturer's protocol was followed from preparation of cell lysate till the detection of active RhoA through western blot analysis. The band intensities were calculated using ImageJ and presented as hybrid-scatter bar graph. (**A**) The image of western blot represents the level of active RhoA in the GTP-bound GTPase pulldown sample (equal volume loaded in each lane). (**B**) The image of western blot represents the level of total RhoA in the whole cell lysate. The band intensity of the blots were quantitated and represented as scatter bar graph. N=2 independent experiments.

Images of full uncut western blot membrane used in the manuscript

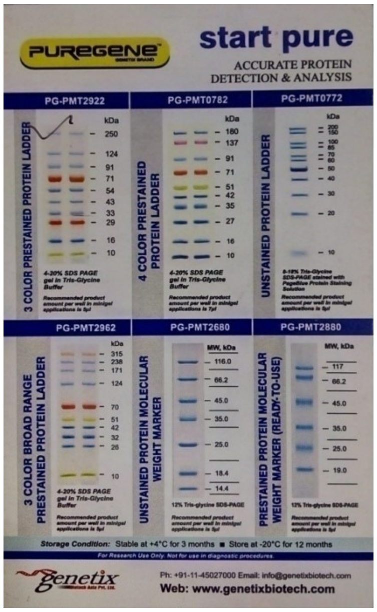

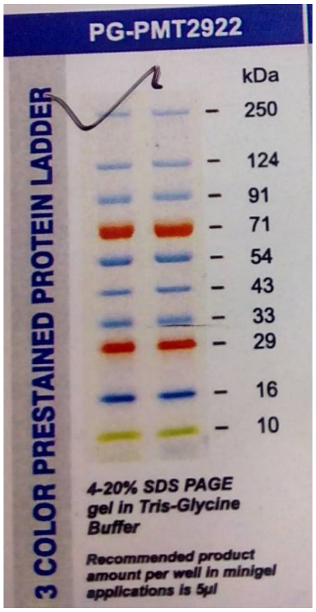

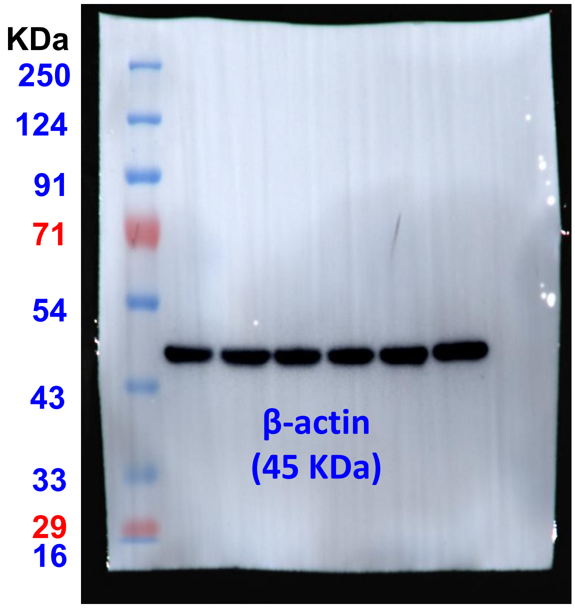

Protein marker cat: **PG-PMT2922;** Vendor: **Puregene**

Fig. 3B

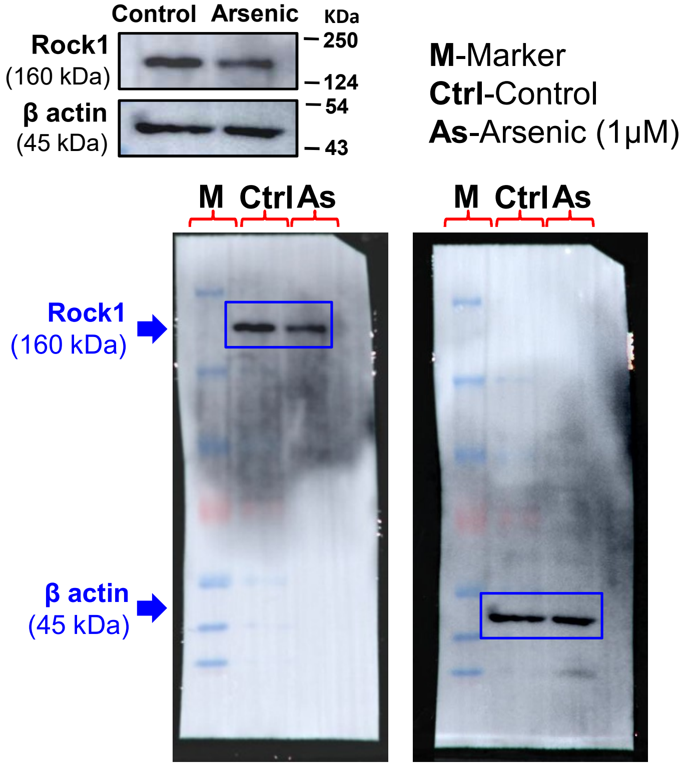

Fig. 3D

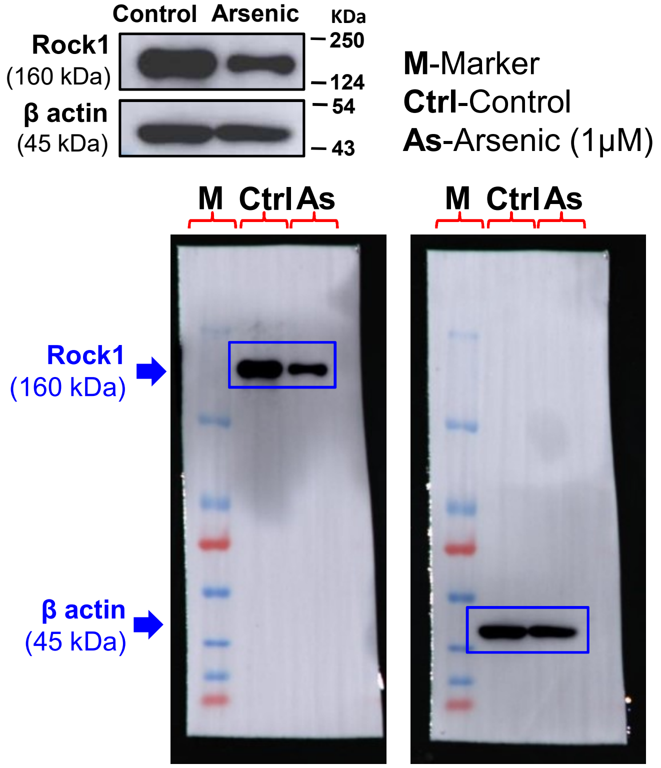

Fig. 3F

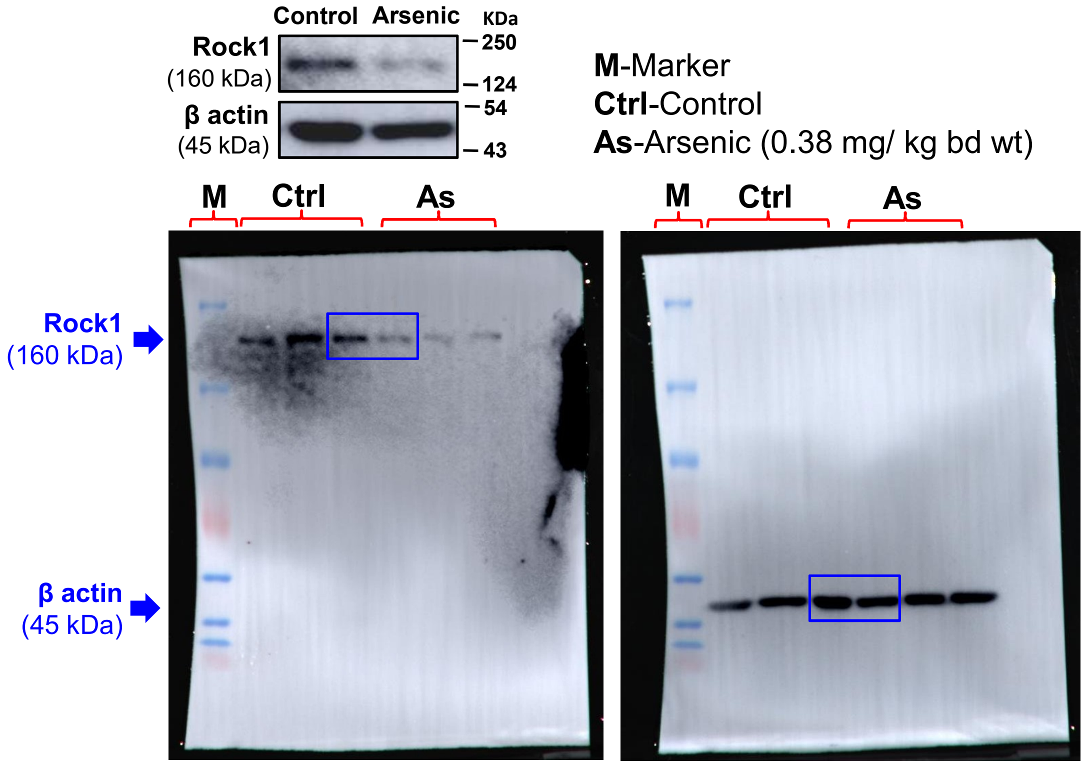

Fig. 4C

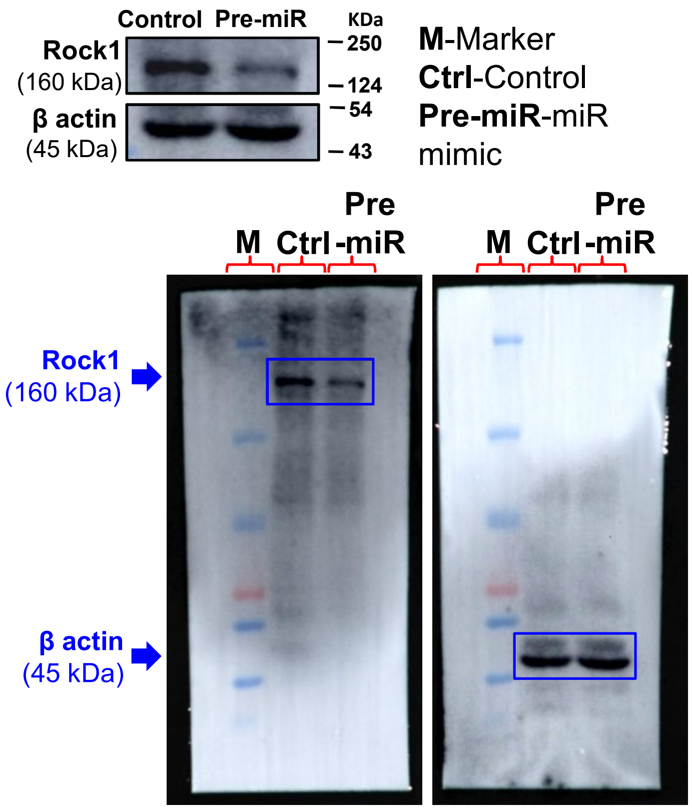

Fig. 4D

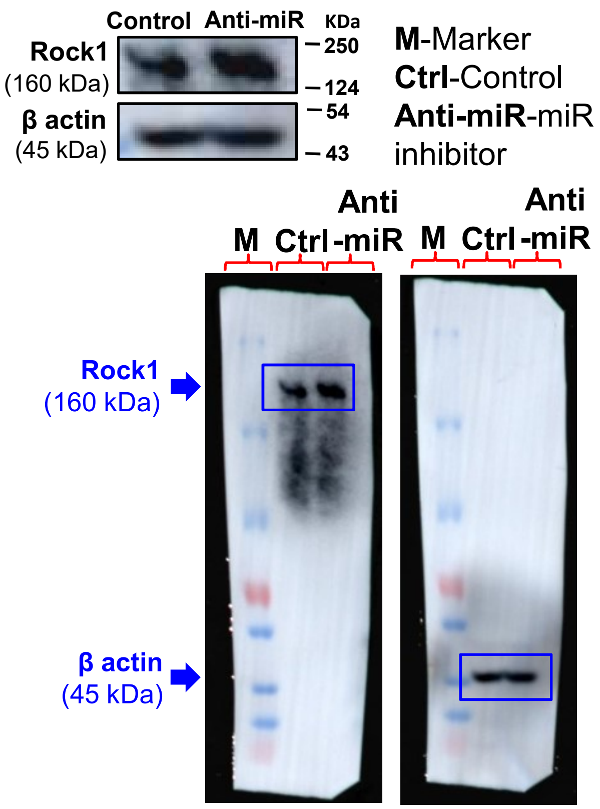

Fig. 4E

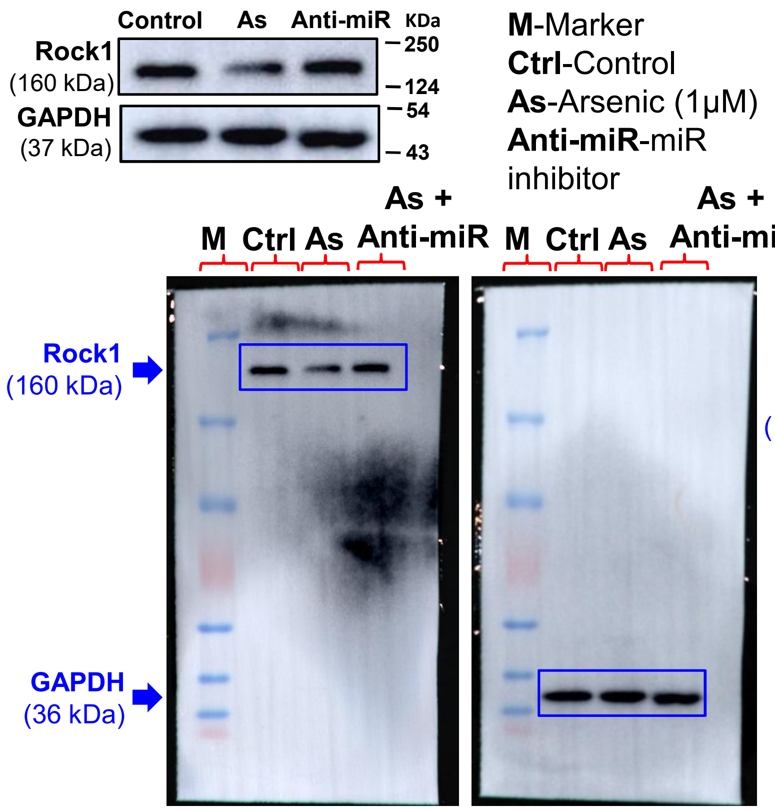

Fig. 4G

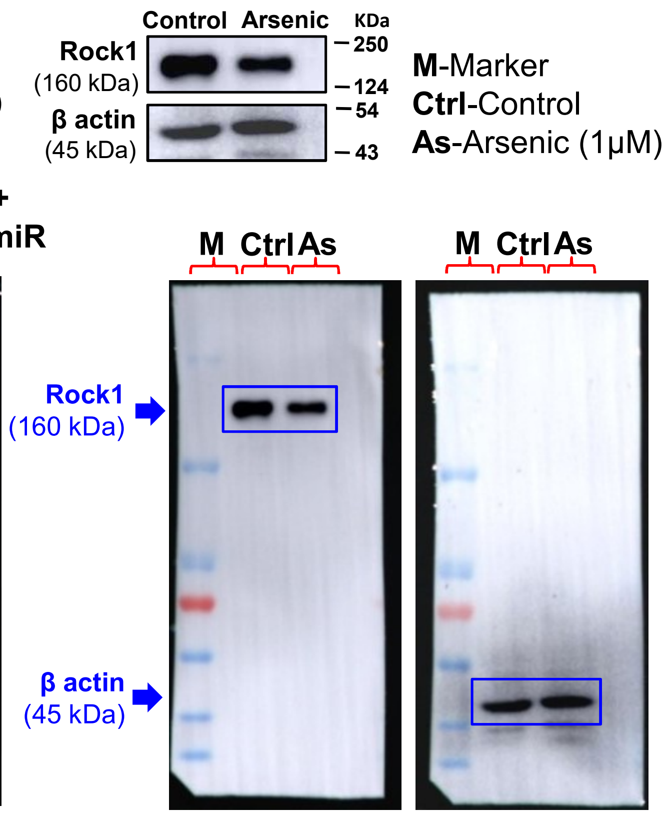

Fig. 7C

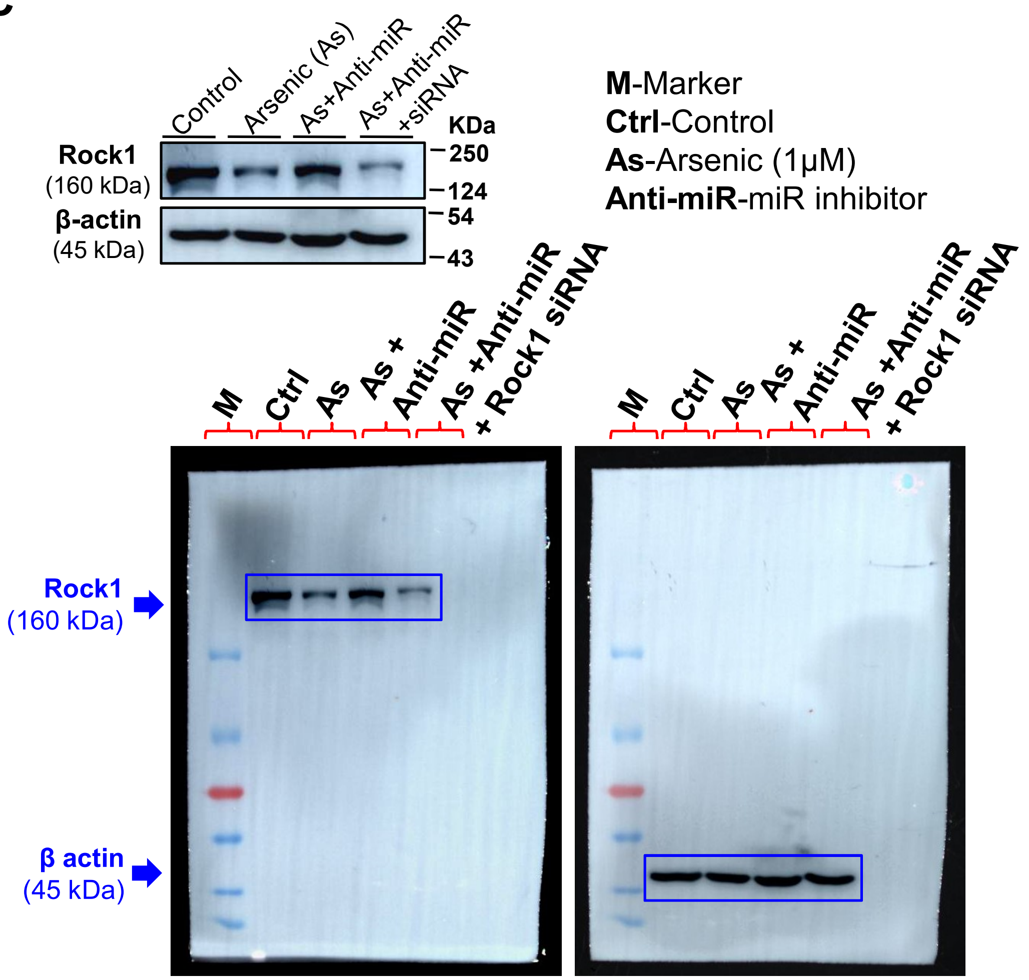

Control
Arsenic (As)
As+Anti-miR
As+Anti-miR +siRNA
KDa
Rock1 (160 kDa)
β-actin (45 kDa)
250
124
54
43
M-Marker
Ctrl-Control
As-Arsenic (1μM)
Anti-miR-miR inhibitor
M
Ctrl
As
As + Anti-miR
As +Anti-miR + Rock1 siRNA
Rock1 (160 kDa)
β actin (45 kDa)

Fig. 7F

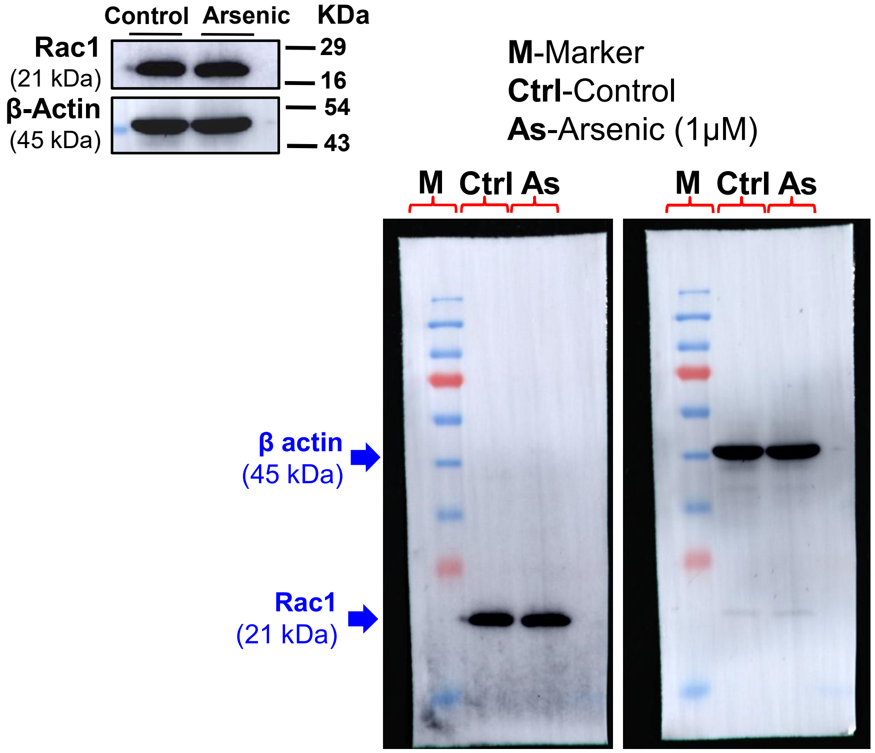

Control
Arsenic
KDa
Rac1 (21 kDa)
β-Actin (45 kDa)
29
16
54
43
M-Marker
Ctrl-Control
As-Arsenic (1μM)
M Ctrl As
M Ctrl As
β actin (45 kDa)
Rac1 (21 kDa)

Fig. 7H

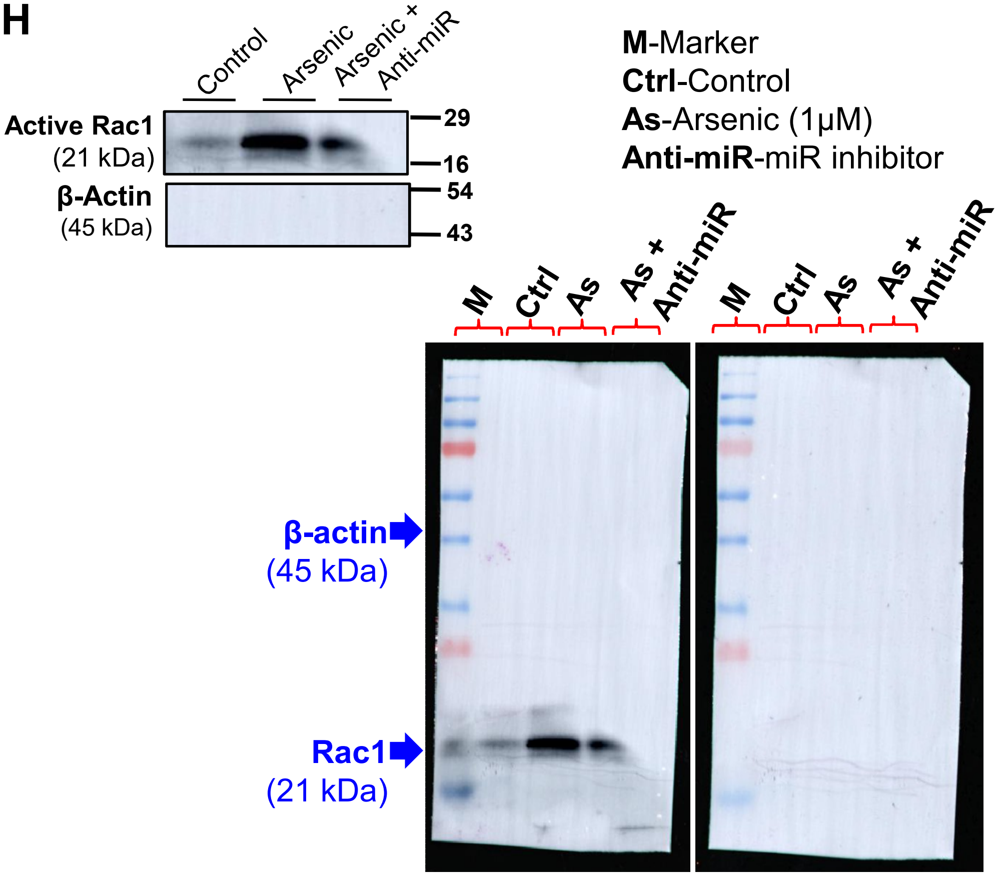
Control
Arsenic
Arsenic +
Anti-miR
Active Rac1
(21 kDa)
β-Actin
(45 kDa)
29
16
54
43
M-Marker
Ctrl-Control
As-Arsenic (1μM)
Anti-miR-miR inhibitor
M
Ctrl
As
As +
Anti-miR
β-actin
(45 kDa)
Rac1
(21 kDa)

Fig. 7I

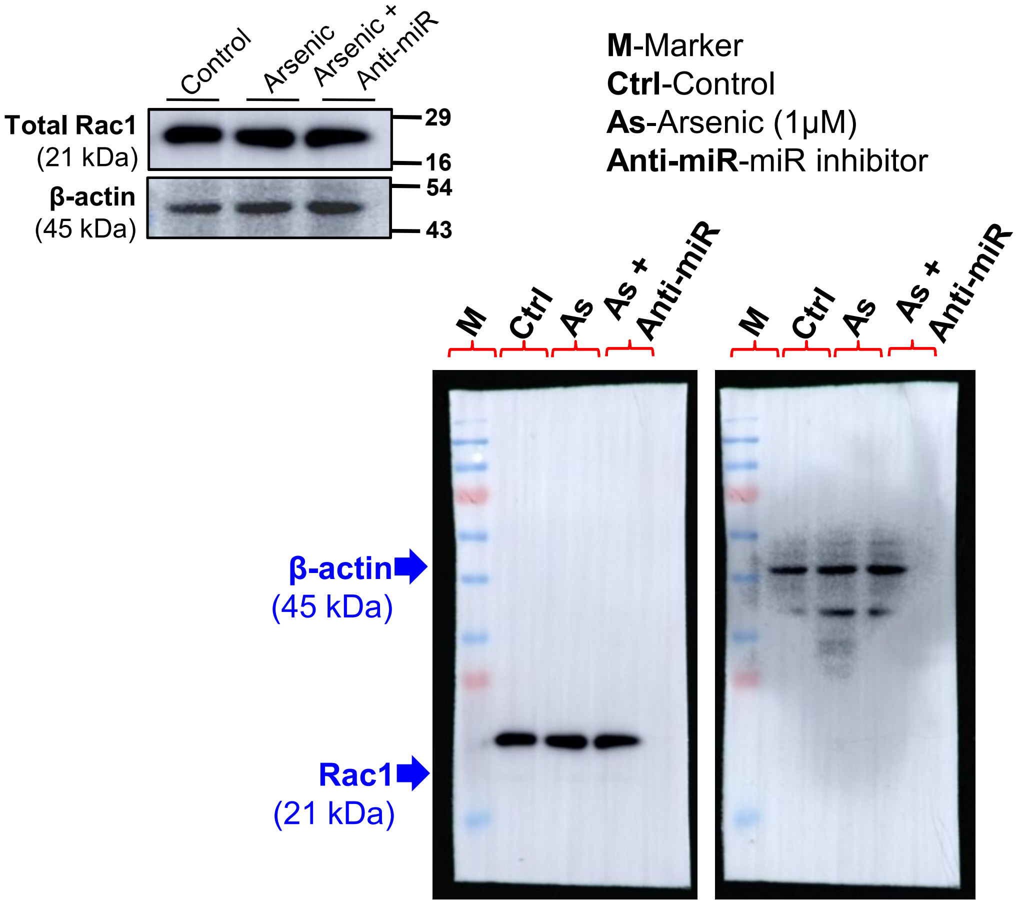
Control
Arsenic
Arsenic +
Anti-miR
Total Rac1
(21 kDa)
β-actin
(45 kDa)
29
16
54
43
M-Marker
Ctrl-Control
As-Arsenic (1μM)
Anti-miR-miR inhibitor
M
Ctrl
As
As +
Anti-miR
β-actin
(45 kDa)
Rac1
(21 kDa)

Fig. S1

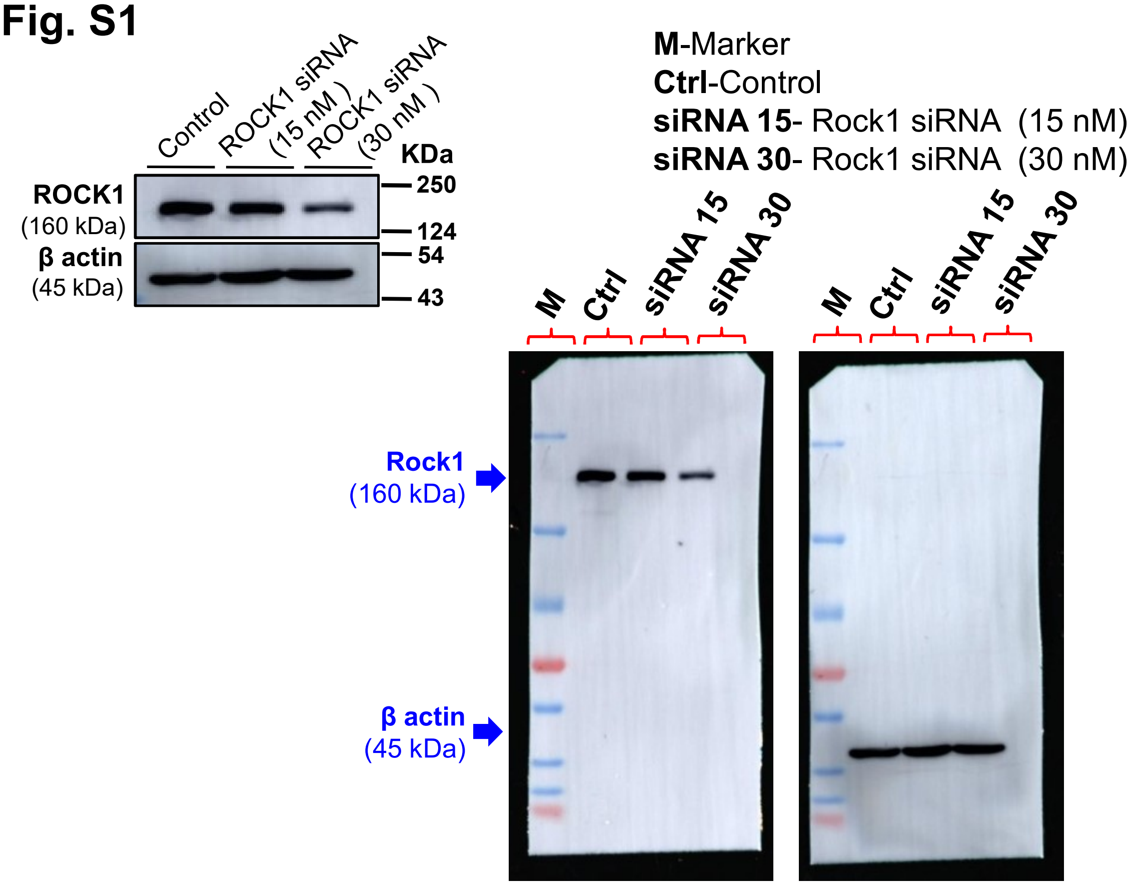

Control
ROCK1 siRNA (15 nM)
ROCK1 siRNA (30 nM)
KDa
ROCK1 (160 kDa)
β actin (45 kDa)
250
124
54
43
M-Marker
Ctrl-Control
siRNA 15- Rock1 siRNA (15 nM)
siRNA 30- Rock1 siRNA (30 nM)
M
Ctrl
siRNA 15
siRNA 30
M
Ctrl
siRNA 15
siRNA 30
Rock1 (160 kDa)
β actin (45 kDa)

Fig. S2

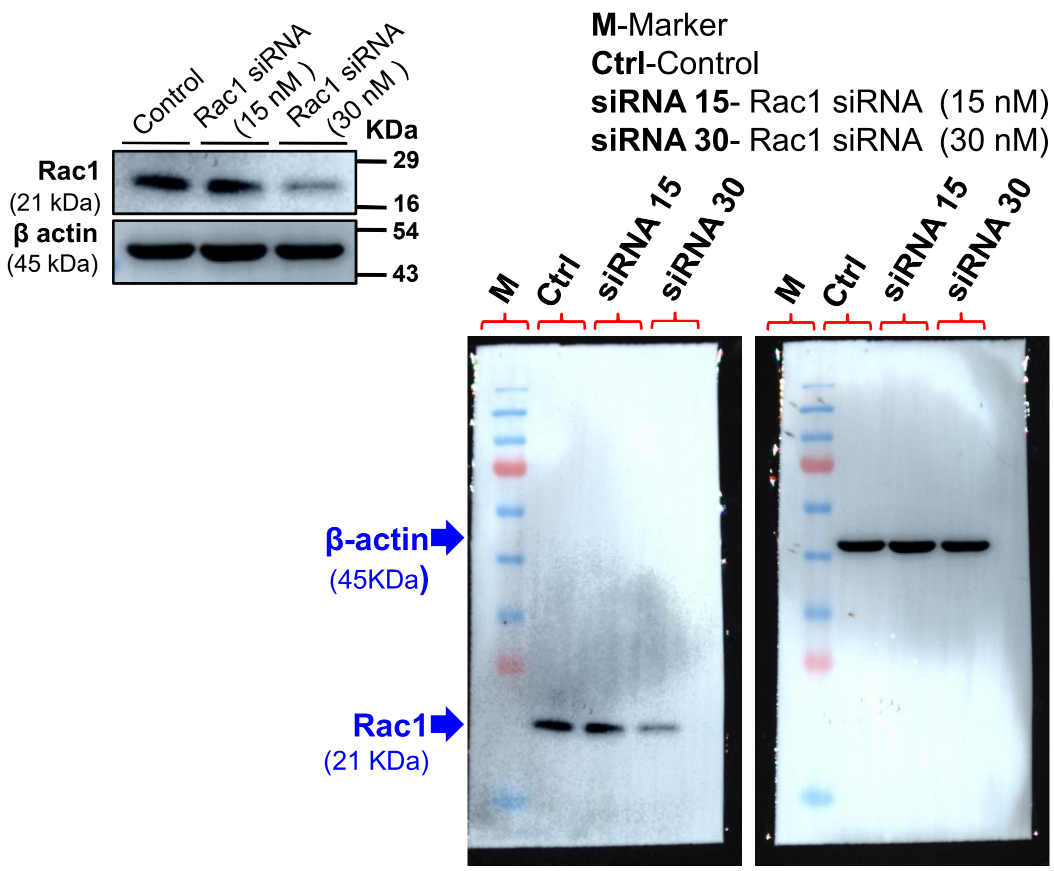

Control
Rac1 siRNA (15 nM)
Rac1 siRNA (30 nM)
KDa
Rac1 (21 kDa)
β actin (45 kDa)
29
16
54
43
M-Marker
Ctrl-Control
siRNA 15- Rac1 siRNA (15 nM)
siRNA 30- Rac1 siRNA (30 nM)
M
Ctrl
siRNA 15
siRNA 30
M
Ctrl
siRNA 15
siRNA 30
β-actin (45KDa)
Rac1 (21 KDa)

Fig. S3 A

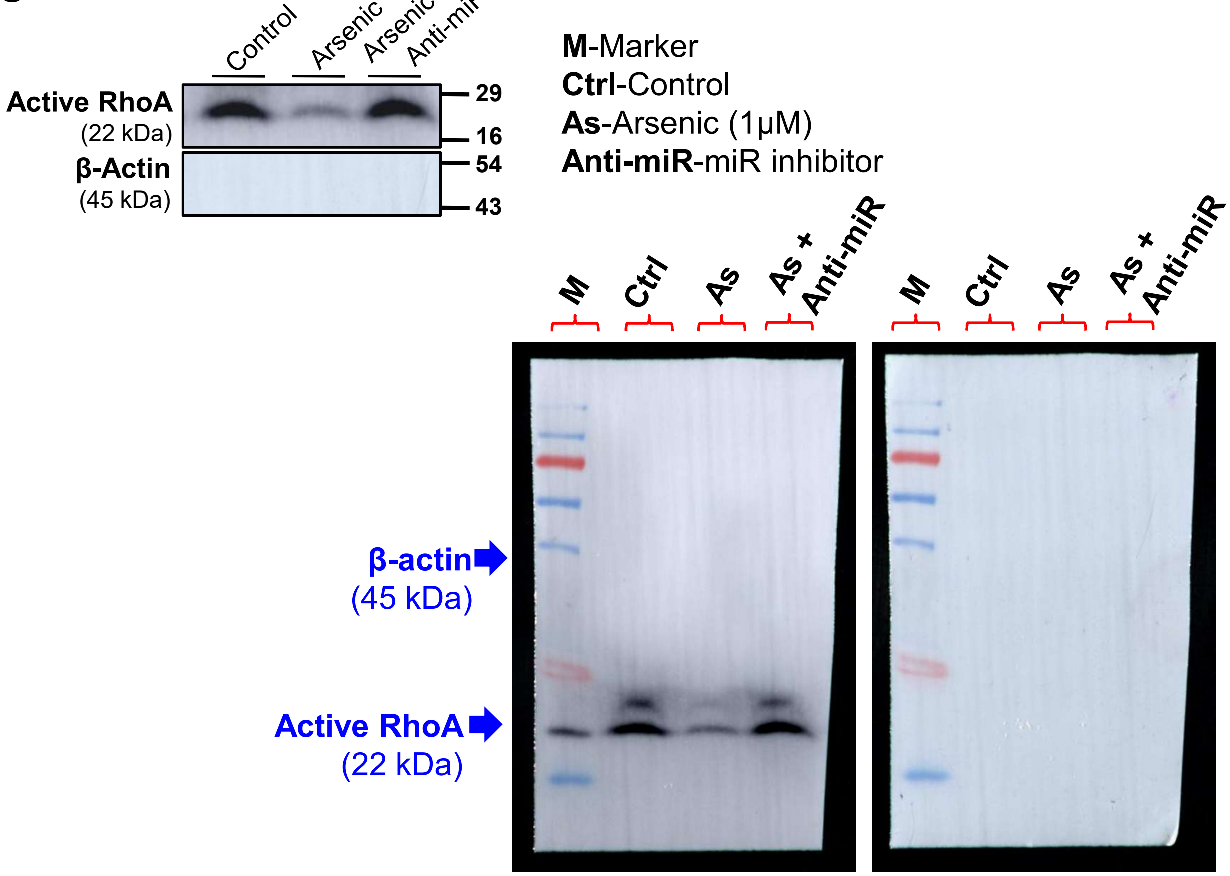

Fig. S3 B

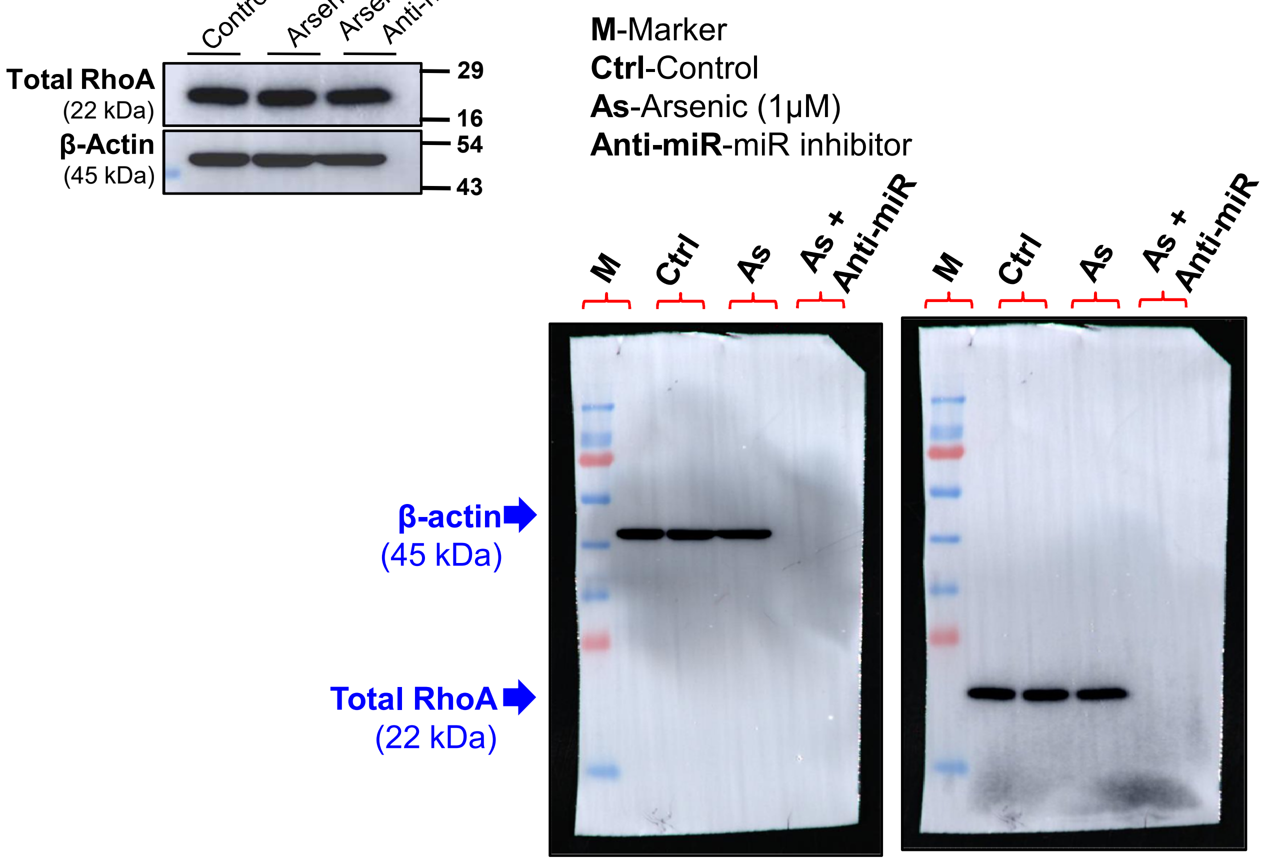

Statistical summary and “*P*” values

Figure-1

**Fig. 1A Mann Whitney test**	
P value	0.0079
Exact or approximate P value?	Exact
P value summary	**
Significantly different (P < 0.05)?	Yes
One- or two-tailed P value?	Two-tailed
Sum of ranks in column A,B	15 , 40
Mann-Whitney U	0

**Fig. 1C Mann Whitney test**	
P value	<0.0001
Exact or approximate P value?	Approximate
P value summary	****
Significantly different (P < 0.05)?	Yes
One- or two-tailed P value?	Two-tailed
Sum of ranks in column A,B	21668 , 59336
Mann-Whitney U	1768

**Fig. 1D Mann Whitney test**	
P value	<0.0001
Exact or approximate P value?	Exact
P value summary	****
Significantly different (P < 0.05)?	Yes
One- or two-tailed P value?	Two-tailed
Sum of ranks in column A,B	1097 , 1459
Mann-Whitney U	236

**Fig.1F ANOVA summary**	
F	6.119
P value	0.0244
P value summary	*
Significant diff. among means (P < 0.05)?	Yes
R square	0.6047

**Tukey's multiple comparisons test**	**Significant?**	**Summary**	**Adjusted P Value**	
Control vs. Arsenic (1µM)	Yes	*	0.0438	A-B
Control vs. LPS (100ng/ml)	Yes	*	0.0386	A-C
Arsenic (1µM) vs. LPS (100ng/ml)	No	ns	0.9500	B-C

Fig. 1G ANOVA summary				
F			28.0	
P value			0.0001	
P value summary			***	
Significant diff. among means (P < 0.05)?			Yes	
R square			0.862	
**Tukey's multiple comparisons test**	**Significant?**	**Summary**	**Adjusted P Value**	
Control vs. Arsenic (1μM)	Yes	***	0.0002	A-B
Control vs. LPS (100ng/ml)	Yes	***	0.0004	A-C
Arsenic (1μM) vs. LPS (100ng/ml)	No	ns	0.9080	B-C

Fig. 1I ANOVA summary				
F			13.07	
P value			0.0022	
P value summary			**	
Significant diff. among means (P < 0.05)?			Yes	
R square			0.7438	
**Tukey's multiple comparisons test**	**Significant?**	**Summary**	**Adjusted P Value**	
Control vs. Arsenic (500 nM)	Yes	**	0.0048	A-B
Control vs. LPS (100ng/ml)	Yes	**	0.0037	A-C
Arsenic (500 nM) vs. LPS (100ng/ml)	No	ns	0.9822	B-C

Fig.1J ANOVA summary				
F			78.5	
P value			<0.0001	
P value summary			****	
Significant diff. among means (P < 0.05)?			Yes	
R square			0.952	
**Tukey's multiple comparisons test**	**Significant?**	**Summary**	**Adjusted P Value**	
Control vs. Arsenic (1μM)	Yes	****	<0.0001	A-B
Control vs. LPS (100ng/ml)	Yes	****	<0.0001	A-C
Arsenic (1μM) vs. LPS (100ng/ml)	No	ns	0.6424	B-C

Figure-2

Fig. 2A ANOVA summary				
F				92.86
P value				<0.0001
P value summary				****
Significant diff. among means (P < 0.05)?				Yes
R square				0.9721
**Tukey's multiple comparisons test**	**Significant?**	**Summary**	**Adjusted P Value**	
Control vs. Arsenic (As,1μM)	Yes	****	<0.0001	A-B
Control vs. As + Rock1 siRNA	Yes	****	<0.0001	A-C
Control vs. Rock1 siRNA (30nM)	Yes	****	<0.0001	A-D
Arsenic (As,1μM) vs. As + Rock1 siRNA	Yes	**	0.0022	B-C
Arsenic (As,1μM) vs. Rock1 siRNA (30nM)	Yes	**	0.0077	B-D
As + Rock1 siRNA vs. Rock1 siRNA (30nM)	No	ns	0.7425	C-D

Fig 2B ANOVA summary				
F				34.63
P value				<0.0001
P value summary				****
Significant diff. among means (P < 0.05)?				Yes
R square				0.8965
**Tukey's multiple comparisons test**	**Significant?**	**Summary**	**Adjusted P Value**	
Control vs. Arsenic (As, 1μM)	No	ns	0.9556	A-B
Control vs. As+Rock1 siRNA	Yes	****	<0.0001	A-C
Control vs. Rock1 siRNA (30nM)	Yes	***	0.0002	A-D
Arsenic (As, 1μM) vs. As+Rock1 siRNA	Yes	****	<0.0001	B-C
Arsenic (As, 1μM) vs. Rock1 siRNA (30nM)	Yes	***	0.0001	B-D
As+Rock1 siRNA vs. Rock1 siRNA (30nM)	No	ns	0.6076	C-D

Figure-3

Fig. 3A Mann Whitney test	
P value	0.3143
Exact or approximate P value?	Exact
P value summary	ns
Significantly different (P < 0.05)?	No
One- or two-tailed P value?	Two-tailed
Sum of ranks in column A,B	22 , 14
Mann-Whitney U	4

Fig. 3B Mann Whitney test	
P value	0.0286
Exact or approximate P value?	Exact
P value summary	*
Significantly different (P < 0.05)?	Yes
One- or two-tailed P value?	Two-tailed
Sum of ranks in column A,B	26 , 10
Mann-Whitney U	0

Fig. 3C Mann Whitney test	
P value	0.3143
Exact or approximate P value?	Exact
P value summary	ns
Significantly different (P < 0.05)?	No
One- or two-tailed P value?	Two-tailed
Sum of ranks in column A,B	22 , 14
Mann-Whitney U	4

Fig. 3D Mann Whitney test	
P value	0.0286
Exact or approximate P value?	Exact
P value summary	*
Significantly different (P < 0.05)?	Yes
One- or two-tailed P value?	Two-tailed
Sum of ranks in column A,B	26 , 10
Mann-Whitney U	0

Fig. 3E Mann Whitney test	
P value	>0.9999
Exact or approximate P value?	Exact
P value summary	ns
Significantly different (P < 0.05)?	No
One- or two-tailed P value?	Two-tailed
Sum of ranks in column A,B	39 , 39
Mann-Whitney U	18

Fig. 3F Mann Whitney test	
P value	0.0022
Exact or approximate P value?	Exact
P value summary	**
Significantly different (P < 0.05)?	Yes
One- or two-tailed P value?	Two-tailed
Sum of ranks in column A,B	57 , 21
Mann-Whitney U	0

Fig. 3G Mann Whitney test	
P value	0.4848
Exact or approximate P value?	Exact
P value summary	ns
Significantly different (P < 0.05)?	No
One- or two-tailed P value?	Two-tailed
Sum of ranks in column A,B	44 , 34
Mann-Whitney U	13

Fig. 3H Mann Whitney test	
P value	<0.0001
Exact or approximate P value?	Exact
P value summary	****
Significantly different (P < 0.05)?	Yes
One- or two-tailed P value?	Two-tailed
Sum of ranks in column A,B	5829 , 3624
Mann-Whitney U	1413

Figure-4

Fig. 4B Mann Whitney test	
P value	0.0286
Exact or approximate P value?	Exact
P value summary	*
Significantly different (P < 0.05)?	Yes
One- or two-tailed P value?	Two-tailed
Sum of ranks in column A,B	10 , 26
Mann-Whitney U	0

Fig. 4C Mann Whitney test	
P value	0.0286
Exact or approximate P value?	Exact
P value summary	*
Significantly different (P < 0.05)?	Yes
One- or two-tailed P value?	Two-tailed
Sum of ranks in column A,B	26 , 10
Mann-Whitney U	0

Fig. 4D Mann Whitney test	
P value	0.0286
Exact or approximate P value?	Exact
P value summary	*
Significantly different (P < 0.05)?	Yes
One- or two-tailed P value?	Two-tailed
Sum of ranks in column A,B	10 , 26
Mann-Whitney U	0

Fig. 4E ANOVA summary				
F	17.64			
P value	0.0008			
P value summary	***			
Significant diff. among means (P < 0.05)?	Yes			
R square	0.7968			
**Tukey's multiple comparisons test**	**Significant?**	**Summary**	**Adjusted P Value**	
Control vs. Arsenic (As, 1μM)	Yes	**	0.0016	A-B
Control vs. As+ Anti-miR-129	No	ns	0.9981	A-C
Arsenic (As, 1μM) vs. As+ Anti-miR-129	Yes	**	0.0015	B-C

Fig. 4F Mann Whitney test	
P value	0.1000
Exact or approximate P value?	Exact
P value summary	ns
Significantly different (P < 0.05)?	No
One- or two-tailed P value?	Two-tailed
Sum of ranks in column A,B	6 , 15
Mann-Whitney U	0

Fig. 4G Mann Whitney test	
P value	0.0286
Exact or approximate P value?	Exact
P value summary	*
Significantly different (P < 0.05)?	Yes
One- or two-tailed P value?	Two-tailed
Sum of ranks in column A,B	26 , 10
Mann-Whitney U	0

Fig. 4H Mann Whitney test	
P value	0.0286
Exact or approximate P value?	Exact
P value summary	*
Significantly different (P < 0.05)?	Yes
One- or two-tailed P value?	Two-tailed
Sum of ranks in column A,B	10 , 26
Mann-Whitney U	0

Figure-5

Fig. 5D ANOVA summary				
F			26.18	
P value			<0.0001	
P value summary			****	
Significant diff. among means (P < 0.05)?			Yes	
R square			0.9197	
**Tukey's multiple comparisons test**	**Significant?**	**Summary**	**Adjusted P Value**	
WT control vs. WT pre-miR-129	Yes	***	0.0002	A-B
WT control vs. Mut1 control	No	ns	>0.9999	A-C
WT control vs. Mut1 pre-miR-129	Yes	****	<0.0001	A-D
WT control vs. Mut2 control	No	ns	>0.9999	A-E
WT control vs. Mut2 pre-miR-129	No	ns	0.9426	A-F
WT control vs. Double Mutant control	No	ns	>0.9999	A-G
WT control vs. Double Mutant pre-miR-129	No	ns	0.9970	A-H
WT pre-miR-129 vs. Mut1 control	Yes	***	0.0002	B-C
WT pre-miR-129 vs. Mut1 pre-miR-129	No	ns	0.4152	B-D
WT pre-miR-129 vs. Mut2 control	Yes	***	0.0002	B-E
WT pre-miR-129 vs. Mut2 pre-miR-129	Yes	****	<0.0001	B-F
WT pre-miR-129 vs. Double Mutant control	Yes	***	0.0002	B-G
WT pre-miR-129 vs. Double Mutant pre-miR-129	Yes	****	<0.0001	B-H
Mut1 control vs. Mut1 pre-miR-129	Yes	****	<0.0001	C-D
Mut1 control vs. Mut2 control	No	ns	>0.9999	C-E
Mut1 control vs. Mut2 pre-miR-129	No	ns	0.9426	C-F
Mut1 control vs. Double Mutant control	No	ns	>0.9999	C-G
Mut1 control vs. Double Mutant pre-miR-129	No	ns	0.9970	C-H
Mut1 pre-miR-129 vs. Mut2 control	Yes	****	<0.0001	D-E
Mut1 pre-miR-129 vs. Mut2 pre-miR-129	Yes	****	<0.0001	D-F
Mut1 pre-miR-129 vs. Double Mutant control	Yes	****	<0.0001	D-G
Mut1 pre-miR-129 vs. Double Mutant pre-miR-129	Yes	****	<0.0001	D-H
Mut2 control vs. Mut2 pre-miR-129	No	ns	0.9426	E-F
Mut2 control vs. Double Mutant control	No	ns	>0.9999	E-G
Mut2 control vs. Double Mutant pre-miR-129	No	ns	0.9970	E-H
Mut2 pre-miR-129 vs. Double Mutant control	No	ns	0.9426	F-G
Mut2 pre-miR-129 vs. Double Mutant pre-miR-129	No	ns	0.9997	F-H
Double Mutant control vs. Double Mutant pre-miR-129	No	ns	0.9970	G-H

Fig. 5F ANOVA summary				
F			11.43	
P value			<0.0001	
P value summary			****	
Significant diff. among means (P < 0.05)?			Yes	
R square			0.7604	
**Tukey's multiple comparisons test**	**Significant?**	**Summary**	**Adjusted P Value**	
Control 0h vs. Control 7h	Yes	**	0.0016	A-B
Control 0h vs. Arsenic 0h	No	ns	>0.9999	A-C
Control 0h vs. Arsenic 7h	No	ns	0.8629	A-D
Control 0h vs. Pre-miR 0h	No	ns	>0.9999	A-E

Control 0h vs. Pre-miR 7h	No	ns	0.3206	A-F
Control 7h vs. Arsenic 0h	Yes	**	0.0016	B-C
Control 7h vs. Arsenic 7h	Yes	***	0.0002	B-D
Control 7h vs. Pre-miR 0h	Yes	**	0.0016	B-E
Control 7h vs. Pre-miR 7h	Yes	****	<0.0001	B-F
Arsenic 0h vs. Arsenic 7h	No	ns	0.8629	C-D
Arsenic 0h vs. Pre-miR 0h	No	ns	>0.9999	C-E
Arsenic 0h vs. Pre-miR 7h	No	ns	0.3206	C-F
Arsenic 7h vs. Pre-miR 0h	No	ns	0.8629	D-E
Arsenic 7h vs. Pre-miR 7h	No	ns	0.9149	D-F
Pre-miR 0h vs. Pre-miR 7h	No	ns	0.3206	E-F

**Fig.5G-Input Mann Whitney test**	
P value	0.0286
Exact or approximate P value?	Exact
P value summary	*
Significantly different (P < 0.05)?	Yes
One- or two-tailed P value?	Two-tailed
Sum of ranks in column A,B	10 , 26
Mann-Whitney U	0

**5G-Pulldown (IP sample) Mann Whitney test**	
P value	0.0286
Exact or approximate P value?	Exact
P value summary	*
Significantly different (P < 0.05)?	Yes
One- or two-tailed P value?	Two-tailed
Sum of ranks in column A,B	10 , 26
Mann-Whitney U	0

**Fig. 5H-Input Mann Whitney test**	
P value	0.3143
Exact or approximate P value?	Exact
P value summary	ns
Significantly different (P < 0.05)?	No
One- or two-tailed P value?	Two-tailed
Sum of ranks in column A,B	14 , 22
Mann-Whitney U	4

**5H-Pulldown (IP sample) Mann Whitney test**	
P value	0.0286
Exact or approximate P value?	Exact
P value summary	*
Significantly different (P < 0.05)?	Yes
One- or two-tailed P value?	Two-tailed
Sum of ranks in column A,B	10 , 26
Mann-Whitney U	0

Figure-6

Fig. 6B ANOVA summary				
F			13.12	
P value			0.0022	
P value summary			**	
Significant diff. among means (P < 0.05)?			Yes	
R square			0.7446	
**Tukey's multiple comparisons test**	**Significant?**	**Summary**	**Adjusted P Value**	
Control vs. Pre-miR-129	Yes	**	0.0050	A-B
Control vs. LPS (100ng/ml)	Yes	**	0.0036	A-C
Pre-miR-129 vs. LPS (100ng/ml)	No	ns	0.9707	B-C

Fig. 6C ANOVA summary				
F			21.15	
P value			0.0004	
P value summary			***	
Significant diff. among means (P < 0.05)?			Yes	
R square			0.8246	
**Tukey's multiple comparisons test**	**Significant?**	**Summary**	**Adjusted P Value**	
Control vs. Pre-miR-129	Yes	***	0.0005	A-B
Control vs. LPS (100ng/ml)	Yes	**	0.0018	A-C
Pre-miR-129 vs. LPS (100ng/ml)	No	ns	0.5798	B-C

Fig. 6E ANOVA summary				
F			6.856	
P value			0.0155	
P value summary			*	
Significant diff. among means (P < 0.05)?			Yes	
R square			0.6037	
**Tukey's multiple comparisons test**	**Significant?**	**Summary**	**Adjusted P Value**	
Control vs. Arsenic(As, 1μM)	Yes	*	0.0173	A-B
Control vs. As + Anti-miR-129	No	ns	0.8033	A-C
Arsenic(As, 1μM) vs. As + Anti-miR-129	Yes	*	0.0464	B-C

Fig. 6F ANOVA summary				
F			27.4	
P value			0.0001	
P value summary			***	
Significant diff. among means (P < 0.05)?			Yes	
R square			0.859	
**Tukey's multiple comparisons test**	**Significant?**	**Summary**	**Adjusted P Value**	
Control vs. Arsenic (As, 1μM)	Yes	***	0.0006	A-B
Control vs. As+Anti-miR-129	No	ns	0.6628	A-C
Arsenic (As, 1μM) vs. As+Anti-miR-129	Yes	***	0.0002	B-C

Fig. 6H ANOVA summary				
F			18.34	
P value			<0.0001	
P value summary			****	
Significant diff. among means (P < 0.05)?			Yes	
R square			0.7098	
**Tukey's multiple comparisons test**	**Significant?**	**Summary**	**Adjusted P Value**	
Control vs. Arsenic (As)	Yes	***	0.0001	A-B
Control vs. As+anti-miR-129	No	ns	0.4976	A-C
Arsenic (As) vs. As+anti-miR-129	Yes	**	0.0010	B-C

Fig. 6J ANOVA summary				
F			182.2	
P value			<0.0001	
P value summary			****	
Significant diff. among means (P < 0.05)?			Yes	
R square			0.4077	
**Tukey's multiple comparisons test**	**Significant?**	**Summary**	**Adjusted P Value**	
Sham control vs. Arsenic (As)	Yes	****	<0.0001	A-B
Sham control vs. As+ Anti-miR-129	Yes	****	<0.0001	A-C
Sham control vs. Anti-miR-129	No	ns	0.6190	A-D
Arsenic (As) vs. As+ Anti-miR-129	Yes	****	<0.0001	B-C
Arsenic (As) vs. Anti-miR-129	Yes	****	<0.0001	B-D
As+ Anti-miR-129 vs. Anti-miR-129	Yes	****	<0.0001	C-D

Fig. 6K ANOVA summary				
F			31.91	
P value			<0.0001	
P value summary			****	
Significant diff. among means (P < 0.05)?			Yes	
R square			0.3603	
**Tukey's multiple comparisons test**	**Significant?**	**Summary**	**Adjusted P Value**	
Sham control vs. Arsenic (As)	Yes	****	<0.0001	A-B
Sham control vs. As+ Anti-miR-129	No	ns	0.3857	A-C
Sham control vs. Anti-miR-129	No	ns	0.9867	A-D
Arsenic (As) vs. As+ Anti-miR-129	Yes	****	<0.0001	B-C
Arsenic (As) vs. Anti-miR-129	Yes	****	<0.0001	B-D
As+ Anti-miR-129 vs. Anti-miR-129	No	ns	0.5444	C-D

Figure-7

Fig. 7A ANOVA summary	
F	13.50
P value	0.0004
P value summary	***
Significant diff. among means (P < 0.05)?	Yes
R square	0.7714

Tukey's multiple comparisons test	Significant?	Summary	Adjusted P Value	
Control vs. Arsenic (As, 1 µM)	Yes	***	0.0010	A-B
Control vs. As+Anti-miR	No	ns	>0.9999	A-C
Control vs. As+Anti-miR+Rock1 siRNA	No	ns	0.9937	A-D
Arsenic (As, 1 µM) vs. As+Anti-miR	Yes	***	0.0009	B-C
Arsenic (As, 1 µM) vs. As+Anti-miR+Rock1 siRNA	Yes	**	0.0015	B-D
As+Anti-miR vs. As+Anti-miR+Rock1 siRNA	No	ns	0.9907	C-D

Fig. 7B ANOVA summary	
F	12.42
P value	0.0022
P value summary	**
Significant diff. among means (P < 0.05)?	Yes
R square	0.8233

Tukey's multiple comparisons test	Significant?	Summary	Adjusted P Value	
Control vs. Arsenic (As, 1µM)	No	ns	0.5937	A-B
Control vs. As+Anti-miR	No	ns	0.9982	A-C
Control vs. As+Anti-miR+Rock1 siRNA	Yes	**	0.0094	A-D
Arsenic (As, 1µM) vs. As+Anti-miR	No	ns	0.5028	B-C
Arsenic (As, 1µM) vs. As+Anti-miR+Rock1 siRNA	Yes	**	0.0020	B-D
As+Anti-miR vs. As+Anti-miR+Rock1 siRNA	Yes	*	0.0116	C-D

Fig. 7C ANOVA summary				
F			37.32	
P value			<0.0001	
P value summary			****	
Significant diff. among means (P < 0.05)?			Yes	
R square			0.9032	
**Tukey's multiple comparisons test**	**Significant?**	**Summary**	**Adjusted P Value**	
Control vs. Arsenic (As, 1µM)	Yes	***	0.0003	A-B
Control vs. As+Anti-miR	No	ns	0.8507	A-C
Control vs. As+Anti-miR+Rock1 siRNA	Yes	****	<0.0001	A-D
Arsenic (As, 1µM) vs. As+Anti-miR	Yes	**	0.0010	B-C
Arsenic (As, 1µM) vs. As+Anti-miR+Rock1 siRNA	Yes	*	0.0458	B-D
As+Anti-miR vs. As+Anti-miR+Rock1 siRNA	Yes	****	<0.0001	C-D

**Fig. 7D ANOVA summary**				
F				19.30
P value				<0.0001
P value summary				****
Significant diff. among means (P < 0.05)?				Yes
R square				0.8284
**Tukey's multiple comparisons test**	**Significant?**	**Summary**	**Adjusted P Value**	
Control vs. Arsenic (As, 1µM)	Yes	**	0.0071	A-B
Control vs. As+Anti-miR	No	ns	0.9414	A-C
Control vs. As+Anti-miR+Rock1 siRNA	Yes	***	0.0004	A-D
Arsenic (As, 1µM) vs. As+Anti-miR	Yes	**	0.0027	B-C
Arsenic (As, 1µM) vs. As+Anti-miR+Rock1 siRNA	No	ns	0.3543	B-D
As+Anti-miR vs. As+Anti-miR+Rock1 siRNA	Yes	***	0.0002	C-D

**Fig. 7E Mann Whitney test**	
P value	0.7000
Exact or approximate P value?	Exact
P value summary	ns
Significantly different (P < 0.05)?	No
One- or two-tailed P value?	Two-tailed
Sum of ranks in column A,B	9 , 12
Mann-Whitney U	3

**Fig. 7F Mann Whitney test**	
P value	0.7000
Exact or approximate P value?	Exact
P value summary	ns
Significantly different (P < 0.05)?	No
One- or two-tailed P value?	Two-tailed
Sum of ranks in column A,B	9 , 12
Mann-Whitney U	3

**Fig. 7G ANOVA summary**				
F				63.63
P value				<0.0001
P value summary				****
Significant diff. among means (P < 0.05)?				Yes
R square				0.9598
**Tukey's multiple comparisons test**	**Significant?**	**Summary**	**Adjusted P Value**	
Control vs. Arsenic (As,1µM)	Yes	***	0.0001	A-B
Control vs. As+Rac1 siRNA	No	ns	0.9360	A-C
Control vs. Rac1 siRNA(30nM)	Yes	**	0.0049	A-D
Arsenic (As,1µM) vs. As+Rac1 siRNA	Yes	****	<0.0001	B-C
Arsenic (As,1µM) vs. Rac1 siRNA(30nM)	Yes	****	<0.0001	B-D
As+Rac1 siRNA vs. Rac1 siRNA(30nM)	Yes	*	0.0103	C-D

Fig.7H ANOVA summary				
F				12.25
P value				<0.0001
P value summary				****
Significant diff. among means (P < 0.05)?				Yes
R square				0.8427
**Tukey's multiple comparisons test**	**Significant?**	**Summary**	**Adjusted P Value**	
Control (Ctrl) vs. Arsenic (As, 1µM)	Yes	**	0.0052	A-B
Control (Ctrl) vs. As+Rac1 inhibitor (10uM)	No	ns	>0.9999	A-C
Control (Ctrl) vs. Rac1 inhibitor (10µM)	No	ns	>0.9999	A-D
Control (Ctrl) vs. Rock1 siRNA	Yes	***	0.0005	A-E
Control (Ctrl) vs. Rock1 siRNA+ Rac1 inhibitor	No	ns	>0.9999	A-F
Control (Ctrl) vs. As+Anti-miR	No	ns	0.9997	A-G
Control (Ctrl) vs. As+Anti-miR+Rac1 inhibitor	No	ns	0.9989	A-H
Arsenic (As, 1µM) vs. As+Rac1 inhibitor (10uM)	Yes	**	0.0034	B-C
Arsenic (As, 1µM) vs. Rac1 inhibitor (10µM)	Yes	**	0.0041	B-D
Arsenic (As, 1µM) vs. Rock1 siRNA	No	ns	0.8949	B-E
Arsenic (As, 1µM) vs. Rock1 siRNA+ Rac1 inhibitor	Yes	*	0.0103	B-F
Arsenic (As, 1µM) vs. As+Anti-miR	Yes	**	0.0022	B-G
Arsenic (As, 1µM) vs. As+Anti-miR+Rac1 inhibitor	Yes	*	0.0157	B-H
As+Rac1 inhibitor (10uM) vs. Rac1 inhibitor (10µM)	No	ns	>0.9999	C-D
As+Rac1 inhibitor (10uM) vs. Rock1 siRNA	Yes	***	0.0003	C-E
As+Rac1 inhibitor (10uM) vs. Rock1 siRNA+ Rac1 inhibitor	No	ns	0.9989	C-F
As+Rac1 inhibitor (10uM) vs. As+Anti-miR	No	ns	>0.9999	C-G
As+Rac1 inhibitor (10uM) vs. As+Anti-miR+Rac1 inhibitor	No	ns	0.9920	C-H
Rac1 inhibitor (10µM) vs. Rock1 siRNA	Yes	***	0.0004	D-E
Rac1 inhibitor (10µM) vs. Rock1 siRNA+ Rac1 inhibitor	No	ns	0.9996	D-F
Rac1 inhibitor (10µM) vs. As+Anti-miR	No	ns	>0.9999	D-G
Rac1 inhibitor (10µM) vs. As+Anti-miR+Rac1 inhibitor	No	ns	0.9960	D-H
Rock1 siRNA vs. Rock1 siRNA+ Rac1 inhibitor	Yes	***	0.0009	E-F
Rock1 siRNA vs. As+Anti-miR	Yes	***	0.0002	E-G
Rock1 siRNA vs. As+Anti-miR+Rac1 inhibitor	Yes	**	0.0013	E-H
Rock1 siRNA+ Rac1 inhibitor vs. As+Anti-miR	No	ns	0.9906	F-G
Rock1 siRNA+ Rac1 inhibitor vs. As+Anti-miR+Rac1 inhibitor	No	ns	>0.9999	F-H
As+Anti-miR vs. As+Anti-miR+Rac1 inhibitor	No	ns	0.9649	G-H

Fig. 7I ANOVA summary				
F				16.64
P value				0.0003
P value summary				***
Significant diff. among means (P < 0.05)?				Yes
R square				0.7350
**Tukey's multiple comparisons test**	**Significant?**	**Summary**	**Adjusted P Value**	
Control vs. Arsenic (As, 1μM)	Yes	***	0.0006	A-B
Control vs. As+anti-miR	No	ns	0.9195	A-C
Arsenic (As, 1μM) vs. As+anti-miR	Yes	**	0.0012	B-C

Fig. 7J ANOVA summary				
F				0.05274
P value				0.9488
P value summary				ns
Significant diff. among means (P < 0.05)?				No
R square				0.008714
**Tukey's multiple comparisons test**	**Significant?**	**Summary**	**Adjusted P Value**	
Control vs. Arsenic (As, 1μM)	No	ns	0.9938	A-B
Control vs. As+anti-miR	No	ns	0.9754	A-C
Arsenic (As, 1μM) vs. As+anti-miR	No	ns	0.9457	B-C
